# Exploring the temporal shift in menstrual hygiene practices among young women across India: a micro and macro perspectives

**DOI:** 10.3389/frph.2025.1532178

**Published:** 2025-07-30

**Authors:** S. K. Singh, Bharti Singh

**Affiliations:** ^1^ National Family Health Survey, International Institute for Population Sciences (IIPS), Mumbai, India; ^2^ Research and Data Analytics, International Institute for Population Sciences (IIPS), Mumbai, India

**Keywords:** menstrual hygiene, young women, reproductive health, adolescent, socio-economic, NFHS, India

## Abstract

**Background:**

Lack of menstrual hygiene practices (MHP) is one of the primary causes of reproductive morbidities among young women. The recent National Family Health Survey (NFHS) showed a significant increase in the use of hygienic menstrual methods in India. This paper aims to investigate the spatial and temporal changes in the prevalence of hygienic menstrual practices from 2016 to 2021, considering micro and macro perspectives.

**Method:**

The study is based on women aged 15–24. The Datasets used in this study are from two recent rounds of the National Family Health Survey, NFHS-4 and NFHS-5. Descriptive, bivariate, multilevel, spatial, and Fairlie decomposition methods have been used to analyze spatial and temporal changes in MHP.

**Results:**

The study illustrates that the prevalence of MHP has increased by 20% points over the past five years, with a significant rise in the use of sanitary napkins, even among marginalized groups. Spatial variation and temporal changes reveal the influence of geospatial attributes, awareness, education, sanitation, and economic prosperity on MHP. Multilevel analysis portrays the maximum clustering in the MHP at the household level in both survey years. Further, Fairlie decomposition reveals that media exposure, followed by the educational attainment of women, contributes highest to the increase in MHP from NFHS-4 to NFHS-5.

**Conclusion:**

The findings of the study present a significant amount of influence of geospatial attributes, including culture and tradition. The extent of “awareness” regarding menstrual hygiene emerged as the most critical driver of escalating MHP in the country. Therefore, addressing socio-economic disparities and implementing interventions through community-level programs, preferably by adopting peer-based approaches with the active participation of self-help groups and frontline workers, is necessary to ensure universal access to sanitary methods.

## 1 Introduction

Menstruation is a vital sign of reproductive health, yet it is taboo in many countries (1–5). Poor menstrual hygiene practices (MHP) have far-reaching adverse effects on the individual's reproductive health, leading to premature birth, stillbirths, miscarriages, infertility problems, reproductive tract infections (RTI), and urinary tract infections (UTI) (6–9). Approximately twenty-six percent of the global population is of reproductive age, and among them, five hundred million individuals lack access to adequate menstrual hygiene facilities (10, 11). Whereas, twenty three percent Indian women age 15–24 have poor MHP (12).

MHP encompasses absorbent types, frequency of changing the absorbent, washing, handling reusable pads/cloths, and other contextual factors (13–15). There are growing evidences that socio-economic and demographic status play a significant role in determining an individual's ability to adopt these practices (2, 16, 17). Another set of literature shows that poor MHP is one of the major causes of reproductive morbidity (2, 8, 9). An Indian study revealed that school-going girls in rural areas who use and reuse old cloths or cloth boiled in water are more prone to RTI (13). In Odisha, India, a case-control study found that UTIs are more common in menstruating women who use reusable absorbents (6). Another study reported that vaginitis was twice as prevalent in women who did not practice hygienic menstrual methods (7).

Furthermore, menstruators face stigma, harassment, and social exclusion, which are primarily due to gender inequality, societal norms, cultural taboos, poverty, inadequate knowledge of menstrual health, and hygiene (18–20). These adversaries also hinder access to the materials and facilities they acquire during menstruation (21). A study based on the Indian scenario reinforces that empowering women is essential in accelerating the level of practising hygienic menstrual methods and providing assistance against various malpractices and taboos (17).

Despite the crucial role of menstruation in reproductive health, it is not explicitly mentioned in the Sustainable Development Goals (SDG). However, it functions as a bridge to achieve several SDG targets. Infographics created by SIMAVI, PATH, and WASH United demonstrate that MHP is linked to SDG 3 (ensure healthy lives and promote wellbeing for all at all ages), SDG 4 (ensure inclusive and equitable quality education and promote lifelong learning), SDG 5 (achieve gender equality and empower all women and girls), SDG 6 (ensure availability and sustainable management of water and sanitation for all), and SDG 8 (decent work and Economic Growth) (22).

While several studies have investigated MHP among either rural or urban women (23–26). Some of the studies comprehensively integrate spatial and multilevel methods to identify the clustering of MHP. However, these approaches have been constrained to specific contexts and often lack a temporal analysis (27, 28). Furthermore, prior studies have not adequately analysed the socio-economic variables contributing to disparities, especially across NFHS-4 to NFHS-5. Hence, to address these gaps, this study aims to:

1. Examine the spatial clustering of menstrual hygiene practices (MHP) among women aged 15–24 in India using NFHS-4 and NFHS-5 data.
2. Explore the temporal changes in MHP between NFHS-4 and NFHS-5 at both national and sub-national levels.
3. Identify the socio-economic and demographic factors contributing to disparities in MHP across the two survey rounds.
4. Integrate micro (individual-level) and macro (state- or district-level) perspectives to provide a comprehensive understanding of the determinants influencing MHP in India.

This research work offers a broader and more nuanced understanding of the evolving patterns and disparities in menstrual hygiene practices across time and space.

## 2 Methods and material

### 2.1 Data and sample

This study is based on the two rounds of the National Family Health Survey NFHS-4 (2015–16) (29) and NFHS-5 (2019–21) (12) conducted by the International Institute for Population Sciences (IIPS), Mumbai under the Ministry of Health and Family Welfare (MoHFW), Government of India. The NFHS is an Indian version of the Demographic and Health Survey (DHS). Data were collected at the individual (children, mothers, and fathers) and household levels. A stratified two-stage sampling design was implemented in the rural and urban area districts. Within each stratum, villages/blocks were selected from the sampling frame using probability proportional to size (PPS) with implicit stratification based on the percentage of the SC/ST population and female literacy. A total of 247,833 women aged 15–24 from NFHS-4 and 241,180 women from NFHS-5 were selected for this study.


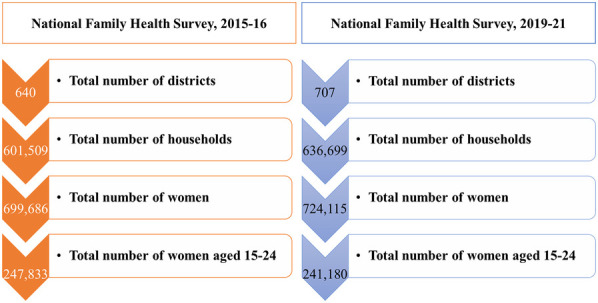


### 2.2 Outcome variable

MHP is the outcome variable in this study. In the NFHS-4, the women's schedule included questions on the various methods used during their menstruation to prevent bloodstains, such as cloths, locally prepared napkins, sanitary napkins, tampons, used nothing at all, and anything else. However, apart from these options, the NFHS-5 also included the use of menstrual cups. These questions were asked from women aged 15–24 years. For this study, the use of locally prepared napkins, sanitary napkins, tampons, and menstrual cups are considered a hygienic method of menstrual protection.

### 2.3 Predictor variable

The predictor variables considered in this study are based on the literature that describes MHP associated with various socio-economic and demographic factors. Thus, the predictors used in this study are the age of the respondent (*15–19 years, 20–24 years*), age at menarche (*7–12 years, 13–17 years, 18–24 years*, knowledge of peak fertility (*No, Yes*) which is used as the proxy variable of reproductive awareness, place of residence (*Rural, Urban*), level of education (*No education, Primary level, Secondary level and Higher level*), religion (*Hindu, Muslim, and Others*), and caste groups (*Scheduled Caste, Scheduled Tribe, Other backward classes, Others*). As NFHS does not provide the “income of the individual”, asset-based “Wealth Quintile” has been used as a proxy of economic status and categorized as “*Poorest*”, “*Poorer*”, “*Middle*”, “*Richer*”, and “*Richest*”. Women who use improved sanitation are defined as the women who reside in such households that have non-shared toilets of the following types: flush/pour flush toilets to piped sewer systems, septic tanks, and pit latrines; ventilated improved pit (VIP)/biogas latrines; pit latrines with slabs; and twin pit/composting toilets. Those who responded at least once a week, read a newspaper or magazine, listened to the radio, watched television or went to the cinema are considered exposed to the mass media.

### 2.4 Statistical analysis

The statistical methods applied in this study consist of two sets. The first set of statistical tools carried out the subnational level analysis, where districts are the unit of analysis. For NFHS-4, a shapefile containing 640 districts of India was generated, whereas for NFHS-5, the number of districts was 707 for spatial analysis. To explore the spatial dependency and clustering of MHP, district-level quintile maps and spatial weights using Queen's contiguity weight matrix were generated.

Furthermore, to identify the interdependency between the use of the hygienic menstrual methods and the selected set of predictor variables, bivariate Moran's I indices, local indicators of spatial autocorrelation (LISA), and spatial autoregression techniques were used to examine the spatial dependence and correlates. Initially, ordinary least squares (OLS) regression was used with MHP, and after that, spatial autoregressive (SAR) models were fitted to the data to decode the spatial effects. Both models were evaluated for the best spatial fit by using diagnostic tests for spatial dependence. A larger value of the Lagrange multiplier (LM) explains better model adequacy. Furthermore, lower AIC values indicate that the spatial error model (SEM) is more suitable for spatial regression (Supplementary Table).

In the second set of statistical analyses at the individual level, descriptive statistics were used to understand the prevalence of MHP, with variation across the socio-economic and demographic characteristics of respondents in NFHS-4 and NFHS-5. Multilevel modelling was applied considering the hierarchical structure of the data, where women were nested within households, households were nested within Primary Sampling Units (PSUs), and PSUs were nested within districts. A three-level random intercept logistic model was specified for the probability of an *i^th^* household in *j^th^* PSU and in the *k^th^* district reporting MHP (Y_ijk_ = 1)

(1) $$\mathrm{Logit}\;(\pi_{\mathrm{ijk}})\;=\;\beta_{o}\;\;+\;\beta X_{\mathrm{ijk}}\;+\;(f_{0k}\;+\;v_{0\mathrm{jk}}\;+\;u_{0\mathrm{ij}})\;\;$$

This model estimates the log odds of $\pi_{ijk}$ adjusted for vector (X_ijk_) of the abovementioned independent variables measured at the household level. Parameter βo represents the log odds of using hygienic menstrual methods belonging to the reference category for all categorical variables. The random effect inside the brackets is interpreted as a residual differential for district k (f_0k_), PSU j (v_0jk_) and household i (u_0ij_), which are assumed to be independent and normally distributed with mean 0 and variance $\sigma_{\;f_{0}}^{2}$, $\sigma_{v_{0}}^{2}$, and $\sigma_{u_{0}}^{2}$, respectively. These variances were quantified between districts and PSU variations in the log odds of using MHP by adopting three different models. Starting with the null model, model 1 included household- and community-level factors, while model 2 added another set of individual-level factors.

Fairlie decomposition has been used to quantify the contributions of various risk factors to the difference in the predicted probabilities of MHP by selected background factors from NFHS-4 to NFHS-5. It can be expressed as follows:

(2) $${\bar{Y}}_{u}-{\bar{Y}}_{r}=\left[\overset{N^{u}}{\underset{i=1}{\sum }}\frac{F(X_{i}^{u}\beta^{u})}{N^{u}}-\overset{N^{r}}{\underset{i=1}{\sum }}\frac{F(X_{i}^{r}\beta^{u})}{N^{r}}\right]+\left[\overset{N^{r}}{\underset{i=1}{\sum }}\frac{F(X_{i}^{r}\beta^{u})}{N^{u}}-\overset{N^{r}}{\underset{i=1}{\sum }}\frac{F(X_{i}^{r}\beta^{r})}{N^{r}}\right]$$

Where, ${\bar{Y}}_{u}$ and ${\bar{Y}}_{r}\;$ represent mean value of MHP at two-time points “u” (2015–16) and “r” (2019–21), “X” represents the set of predictor variables, *β* represents the coefficient, *N^u^* and *N^r^* represent the sample size at time points u and r, respectively. The first term in the equation represents characteristics, and the latter term represents the discrimination effect, that is, the differences caused by various characteristic regression coefficients. Positive coefficient represents positive contribution to the difference and vice-versa (30).

This study was analyzed using STATA Version 17 and Geo-da version 1.20.0.8. All results were derived by applying the sampling weight provided by the Demographic and Health Survey (DHS), India.

## 3 Results

Table 1 shows a significant change in the prevalence of MHP in almost every state and country as a whole, from 2015–16 to 2019–21. It is evident from the results of NFHS-5 that every state in India has experienced a significant increase in the proportion of adolescents and young women aged 15–24 years who practised hygienic methods, except Mizoram. Mizoram shows a decline in MHP from 2015–16 to 2019–21 by four percentage points, where 92% of young women were using sanitary methods of menstrual protection in 2015–16. In contrast, Odisha shows a significant increase of 32 percentage points in MHP from 2015–16 to 2019–21. In the most recent survey NFHS-5 (2019–21), Tamil Nadu was the state with the highest prevalence (98.3%), and Bihar had the lowest prevalence (58.9%). It is noteworthy that Bihar has experienced an increase of 28 percentage points over the past four years. A proportion of young women living in urban areas were significantly more inclined to use hygienic methods of menstrual protection in each state, except in Sikkim, where the urban‒rural divide was almost negligible, primarily due to its more diminutive size.

**Table 1:** Percentage of adolescents and young women aged 15–24 years who reported hygienic menstrual practices in urban and rural areas of different states of India, NFHS-4 (2015–16) & NFHS-5 (2019–21).

States of India	NFHS-4			NFHS-5		
	Urban % (95% CI)	Rural % (95% CI)	Total % (95% CI)	Urban % (95% CI)	Rural % (95% CI)	Total % (95% CI)
India	77.4 (76.6–78.2)	48.1 (47.7–48.6)	57.5 (57.1–58.0)	89.3 (88.8–89.9)	72.3 (71.9–72.6)	77.3 (77.0–77.6)
Andhra Pradesh	77.6 (73.3–81.4)	62.9 (60.2–65.6)	67.5 (65.2–69.7)	90.6 (87.6–93)	82.5 (80.3–84.6)	85.1 (83.3–86.7)
Arunachal Pradesh	78.6 (75–81.9)	71.1 (68.5–73.7)	73.3 (71.2–75.4)	93.5 (91–95.4)	91.4 (90.1–92.5)	91.8 (90.6–92.8)
Assam	70.8 (65.8–75.4)	40.9 (39.2–42.7)	44.8 (43.2–46.4)	83.4 (78.6–87.3)	64.4 (62.6–66.2)	66.9 (65.2–68.6)
Bihar	55.6 (51.5–59.6)	27.3 (26.1–28.5)	31 (29.8–32.2)	74.7 (70.3–78.7)	56.1 (54.7–57.4)	58.9 (57.6–60.2)
Chhattisgarh	72.7 (68.9–76.1)	39.4 (37.3–41.5)	47.1 (45.2–49)	83.2 (78.9–86.7)	64.8 (62.8–66.7)	68.8 (67.1–70.5)
Goa	94.1 (89.3–96.8)	81.7 (72.8–88.1)	89.3 (84.7–92.6)	96.2 (91.5–98.4)	97.6 (92.6–99.2)	96.8 (93.7–98.4)
Gujarat	70 (65.1–74.4)	53.5 (50.8–56.1)	60.3 (57.8–62.7)	78.8 (75–82.1)	58.9 (57.1–60.7)	66.5 (64.7–68.2)
Haryana	82.5 (78.9–85.7)	75.8 (74–77.6)	78.3 (76.6–80)	96.7 (95.6–97.5)	91.6 (90.6–92.5)	93.2 (92.4–93.9)
Himachal Pradesh	90 (80.3–95.2)	83.7 (80.9–86.1)	84.3 (81.7–86.5)	96.3 (90.4–98.6)	90.9 (88.9–92.6)	91.6 (89.8–93.1)
Jammu and Kashmir	85 (81.9–87.6)	60.1 (58.1–62.2)	66.5 (64.7–68.3)	85.9 (81.9–89.1)	70.9 (68.6–73.1)	74.4 (72.4–76.3)
Jharkhand	77.2 (74.5–79.6)	39.4 (37.7–41.2)	49.6 (48.1–51.1)	88.2 (85.3–90.6)	70.8 (69–72.6)	74.9 (73.3–76.4)
Karnataka	81.6 (78.9–84)	62.1 (59.8–64.3)	70.3 (68.5–72)	91 (89.1–92.6)	79.8 (78.1–81.5)	84.2 (82.9–85.5)
Kerala	91.7 (89.5–93.4)	88.5 (86.3–90.3)	90 (88.5–91.3)	94.8 (93–96.2)	91.5 (89.5–93.1)	93.1 (91.8–94.2)
Madhya Pradesh	65.4 (63.3–67.4)	26.4 (25.3–27.5)	37.6 (36.6–38.7)	81.9 (79.5–84.1)	53.4 (52.0–54.8)	60.5 (59.3–61.8)
Maharashtra	77 (73.8–79.8)	55.7 (53.4–58.1)	66.1 (64.1–68.1)	90.3 (88.3–92.1)	80.2 (78.8–81.6)	84.9 (83.7–86)
Manipur	80.5 (76.6–83.9)	73.3 (70.5–75.9)	76.1 (73.8–78.2)	89.6 (86–92.3)	79.9 (76.2–83.1)	83.3 (80.7–85.6)
Meghalaya	85.2 (81.5–88.3)	57.1 (53.6–60.5)	63.7 (60.8–66.6)	85 (80.3–88.8)	59.1 (55.6–62.5)	64.9 (62–67.7)
Mizoram	96.3 (93.8–97.8)	88.8 (85.9–91.2)	93.4 (91.6–94.8)	93.6 (87.7–96.7)	84.6 (80.8–87.7)	89.8 (86.5–92.3)
Nagaland	82 (78.4–85.1)	65.9 (61.6–69.9)	72.4 (69.5–75.2)	87.6 (82.6–91.3)	76.6 (73.4–79.6)	80.4 (77.7–82.8)
Odisha	70 (65.9–73.7)	42.8 (41.2–44.4)	47.4 (45.9–48.9)	91.7 (89.4–93.4)	79.5 (77.9–80.9)	81.5 (80.2–82.8)
Punjab	91.2 (89.2–92.8)	80.7 (78.6–82.6)	84.4 (82.9–85.9)	95.4 (93.7–96.6)	91.9 (90.7–93)	93.2 (92.2–94)
Rajasthan	78.8 (76.5–81)	47.9 (46.3–49.5)	55.2 (53.9–56.5)	92.2 (90.3–93.7)	81.9 (80.7–83)	84.1 (83.1–85)
Sikkim	92.7 (87.4–95.9)	80.8 (76.2–84.6)	84.6 (81.2–87.6)	87.1 (76.6–93.3)	85.7 (81.9–88.8)	86.3 (81.9–89.7)
Tamil Nadu	93.5 (92.1–94.7)	89.5 (88.2–90.6)	91.4 (90.5–92.2)	98.6 (97.8–99.2)	98 (97.3–98.5)	98.3 (97.8–98.7)
Telangana	86.9 (83.5–89.7)	67.2 (64–70.2)	76.6 (74.4–78.7)	97 (95.9–97.8)	90.8 (89.6–91.9)	93.1 (92.2–93.9)
Tripura	56.5 (47.8–64.8)	38.6 (34.5–42.9)	43.5 (39.7–47.4)	83.4 (76.1–88.8)	64.2 (60.9–67.3)	69.1 (66.1–72)
Uttar Pradesh	68.6 (66.7–70.5)	39.9 (39–40.8)	47.1 (46.2–47.9)	86.7 (85.2–88.1)	68.4 (67.6–69.3)	72.6 (71.8–73.3)
Uttarakhand	78.9 (74.8–82.5)	65 (62.6–67.4)	69.9 (67.7–72)	94.5 (91.6–96.5)	89.7 (87.8–91.3)	91.2 (89.7–92.6)
West Bengal	72.9 (68–77.3)	47.6 (45.4–49.9)	54.9 (52.9–57)	91.2 (89.1–92.9)	79.8 (78.2–81.3)	83.1 (81.8–84.3)

CI, confidence interval.

**Figure 1:**
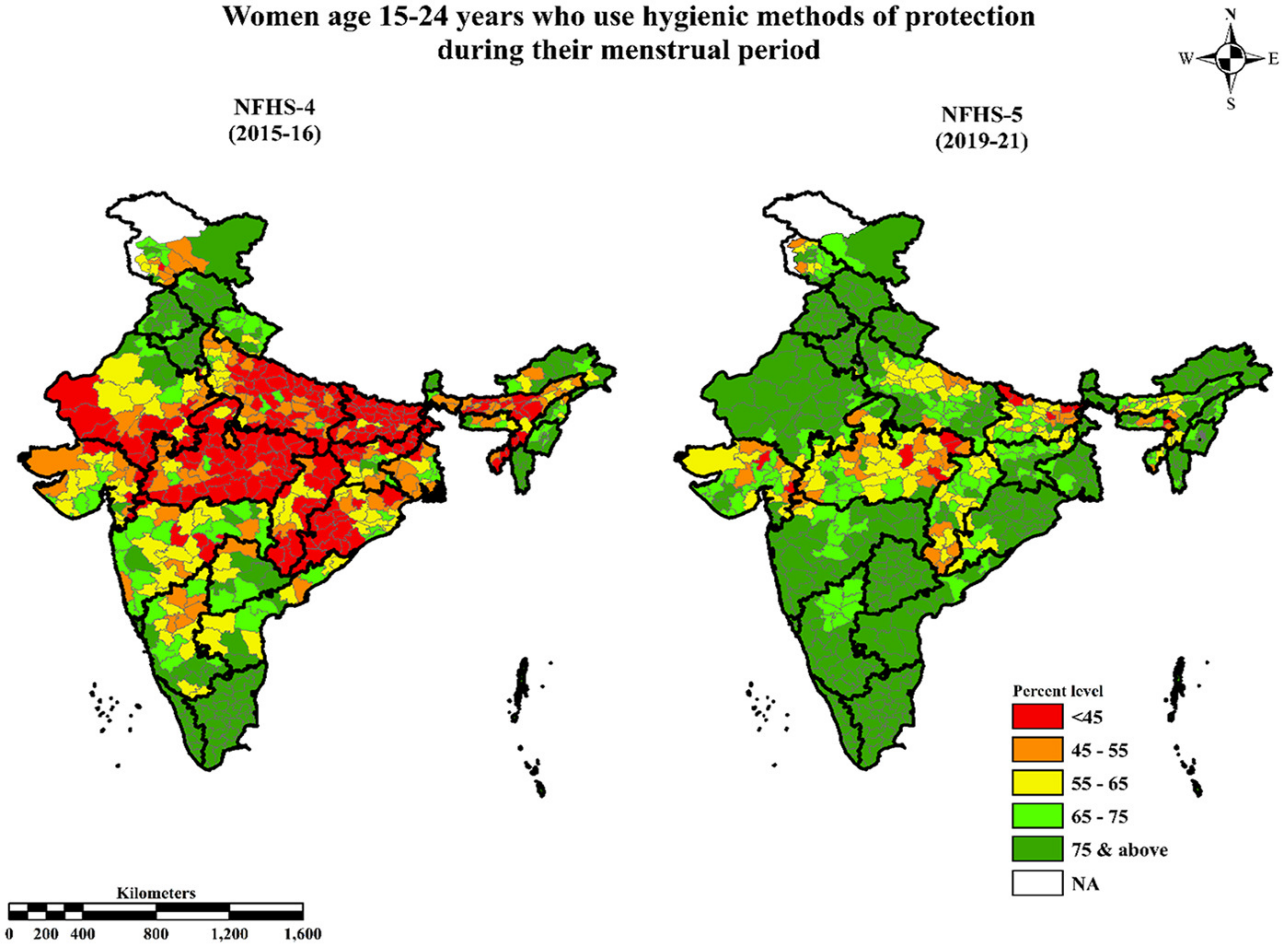
Prevalence of hygienic menstrual practices among adolescents and young women aged 15–24 in different districts of India, NFHS-4 (2015–16) & NFHS-5 (2019–21).

Figure 1 demonstrates the temporal evolution in the prevalence of MHP based on the comparison between NFHS-4 and NFHS-5. It is evident from the map that in 2015–16, a total of 333 out of 640 districts across the country had a prevalence of MHP below sixty percent. Over a span of five years, this number of districts decreased to 99 among 707 districts, indicating a substantial improvement in MHP nationwide. Notably, Central India stands out as a region that has experienced significant positive transformations in MHP during this five-year period. Nevertheless, it continued to have the highest concentration of districts with the lowest prevalence of MHP in both the survey years. Furthermore, districts situated in the southern region of the country showed a higher prevalence of MHP in NFHS-4 and NFHS-5.

**Figure 2:**
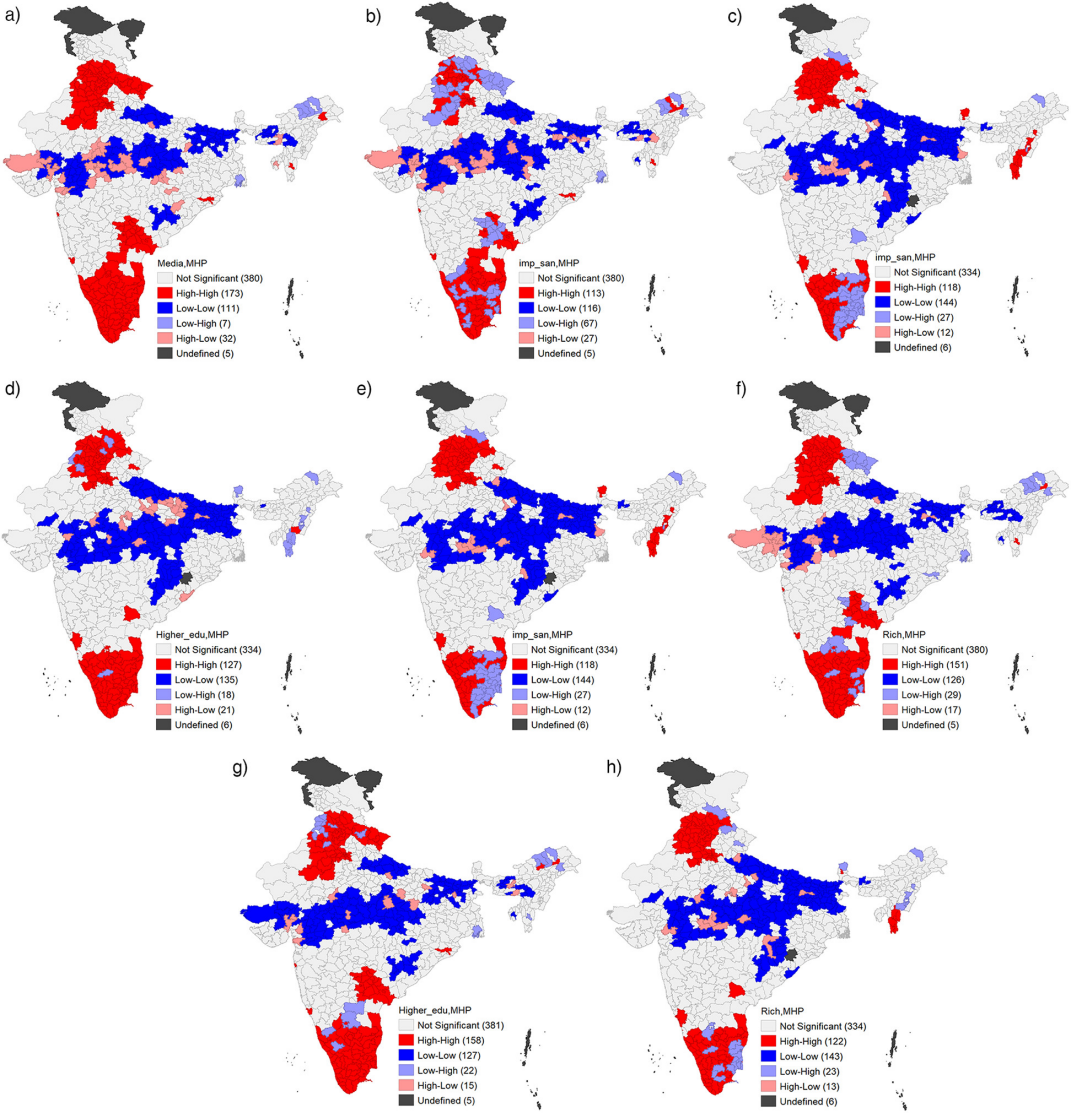
**(A)** Bivariate LISA cluster map of hygienic menstrual practice among adolescents and young women aged 15–24 with their education India, 2015–16 (NFHS 4). **(B)** Bivariate LISA cluster map of hygienic menstrual practice among adolescents and young women aged 15–24 with their wealth status. India, 2015–16 (NFHS 4). **(C)** Bivariate LISA cluster map of hygienic menstrual practice among adolescents and young women aged 15–24 with improved sanitation facility, India, 2015–16 (NFHS 4). **(D)** Bivariate LISA cluster map of hygienic menstrual practice among adolescents and young women aged 15–24 with exposure to media, India, 2015–16 (NFHS 4). **(E)** Bivariate LISA cluster map of hygienic menstrual practice among adolescents and young women aged 15–24 with their education India, 2019–21 (NFHS 5). **(F)** Bivariate LISA cluster map of hygienic menstrual practice among adolescents and young women aged 15–24 with their wealth status. India, 2019–21 (NFHS 5). **(G)** Bivariate LISA cluster map of hygienic menstrual practice among adolescents and young women aged 15–24 with improved sanitation facility, India, 2019–21 (NFHS 5). **(H)** Bivariate LISA cluster map of hygienic menstrual practice among adolescents and young women aged 15–24 with exposure to media, India, 2019–21 (NFHS 5).

The bivariate LISA maps (Figures 2A–H) illustrate the spatial dependency and clustering of MHP with a number of key markers across the districts and changing patterns in clustering in the last five years. These markers are “higher level of education (proportions having 10 or more years of schooling)”, “wealthier class (fourth and fifth wealth quintiles)”, “improved sanitation”, and “exposure to mass media” in NFHS-4 and NFHS-5. Hotspot districts are areas where there is a higher concentration of districts with improved MHP and higher levels of the aforementioned markers. These districts exhibit positive spatial clustering, suggesting that neighbouring districts also tend to have a higher prevalence of MHP under the influence of the socio-cultural environment. In contrast, cold spot districts are areas where both parameters display lower levels. Most of the hotspot districts were concentrated in the southern and northern regions (around Delhi, Punjab, and Uttarakhand) of the country. In contrast, cold spots are located in central India and in some parts of Uttar Pradesh and Bihar. These findings indicate that demographically developed states have the maximum number of hotspot districts with clustering in the use of hygienic methods of menstrual protection.

**Figure 3:**
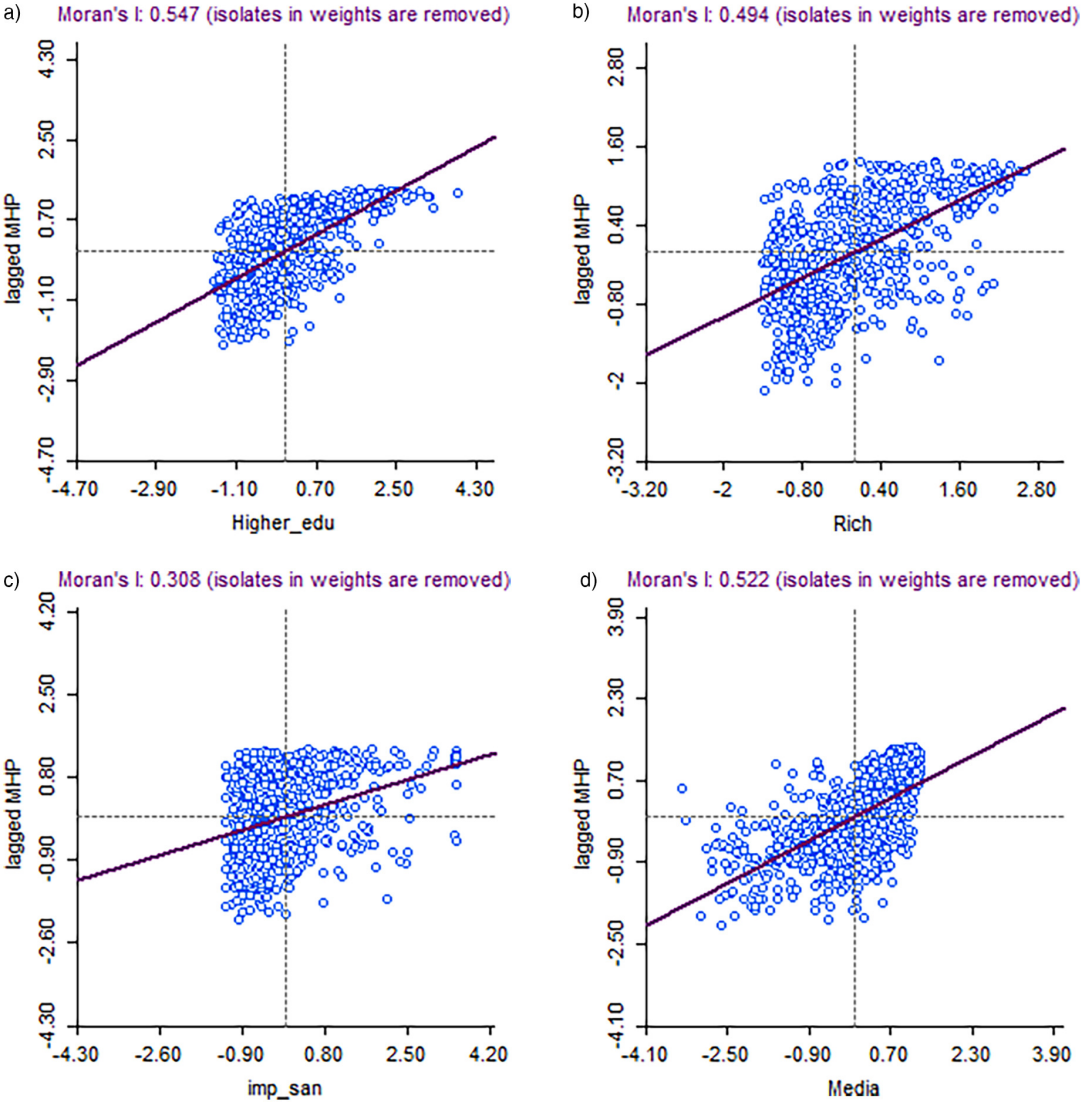
**(A)** Moran's I statistic of spatial autocorrelation of hygienic menstrual practice among adolescents and young women aged 15–24 with their education, India, 2019–21 (NFHS 5). **(B)** Moran's I statistic of spatial autocorrelation of hygienic menstrual practice among adolescents and young women aged 15–24 with their wealth status, India, 2019–21 (NFHS 5). **(C)** Moran's I statistic of spatial autocorrelation of hygienic menstrual practice among adolescents and young women aged 15–24 with improved sanitation, India, 2019–21 (NFHS 5). **(D)** Moran's I statistic of spatial autocorrelation of hygienic menstrual practice among adolescents and young women aged 15–24 with exposure to media, India, 2019–21 (NFHS 5).

Figures 3A–D portrays the value of bivariate Moran's I reflecting quantitative evidence of the interdependency of the outcome variables and the selected predictor variables considering the spatial clustering through significant autocorrelation values. Figure 3A shows that the value of Moran's I statistic is highest (Moran's I = 0.58) in “higher education”, indicating that the women with higher education are more concentrated toward the mean. Districts with more exposure of mass media (Figure 3D) were also more likely to record a higher rate of MHP (Moran's I = 0.52). A higher proportion of women who belonged to economically well-off households (Moran's I = 0.49) also had a significantly higher prevalence of MHP (Figure 3B). Furthermore, the significance maps for all selected variables clearly demonstrated that a substantial number of districts exhibited statistically significant spatial clustering (*p* < 0.05), confirming that the observed global spatial associations were not merely the result of a few extreme values (Supplementary Figures 4A–H). To ensure robustness, we also conducted Local Moran's I analysis with 999 permutations for each predictor. This approach provides a more granular view of spatial patterns and reinforces that spatial outliers do not disproportionately influence the results (Supplementary Figures 5A–D, 6A–D).

Table 2 presents the results of spatial autoregression for MHP by women's education level, exposure to mass media, improved sanitation facility, wealthier class, and urban place of residence for two time periods. The OLS model presents the degree of linear relationship between the independent variables and the dependent variable without considering their spatial dependence. After applying the regression diagnostic test, the spatial error model showed a higher value of lag coefficient (lambda), making it the best-fit model for this study. We observed significant geospatial clustering in the outcome and predictor variables. Concurrently, the study presents the OLS and spatial error models for the two time periods. Higher education was positively and significantly associated with the use of hygienic menstrual methods in both the time periods. However, 2019–21 (NFHS-5) showed a higher degree of association than 2015–16 (NFHS-4). Women exposed to any kind of mass media and those who belonged to the wealthier class were positively and significantly associated with MHP in 2015–16 and 2019–21. However, the degree of association declined over time. Women who did not belong to the SC, ST, and other backward classes had a positive but weak association with MHP in both periods. Women residing in urban areas had no significant effect on their MHP.

**Table 2:** Spatial regression analysis of hygienic menstrual practice among adolescents and young women aged 15–24 in India, NFHS-4 (2015–16) & NFHS-5 (2019–21).

Predictors	NFHS-4		NFHS-5	
	Ordinary least square	Spatial error model	Ordinary least square	Spatial error model
Higher education	0.39 (0.000)	0.21 (0.000)	0.56 (0.000)	0.18 (0.019)
Exposure to media	0.43 (0.000)	0.41 (0.000)	0.37 (0.000)	0.33 (0.000)
Wealth class	0.23 (0.000)	0.26 (0.000)	0.09 (0.000)	0.14 (0.000)
Other caste	0.01 (0.000)	0.09 (0.000)	0.11 (0.000)	0.06 (0.000)
Urban population	0.01 (0.739)	0.01 (0.583)	−0.08 (0.000)	−0.02 (0.328)
Number of observations	640	640	707	707
AIC	5,002.47	4,516.22	5,186	4,795.2
Adjusted R square	0.69	0.87	0.60	0.80
Error lag value (Lambda)		0.81		0.76

Table 3 shows the percent distribution of young women aged 15–24 in India with three different types of absorbents used during menstruation and by background characteristics in 2015–16 and 2019–21. Prevalence of using sanitary napkins has increased by twenty percent in all age groups of the menarche from NFHS-4 to NFHS-5. Women having exposure to mass media show a relatively higher percentage of utilising hygienic methods of menstrual protection. There was an overall 20% point increase in the use of hygienic methods of menstrual protection from NFHS-4 to NFHS-5. A significantly higher proportion of young urban women than their rural counterparts reported using hygienic methods of menstrual protection. Similarly, educational attainment significantly affected the use of hygienic menstrual methods. In the NFHS-4, eighty-six percent of women who had a higher level of education used hygienic methods, which further increased to ninety-five percent in the NFHS-5. The respondent's religion also played a crucial role during NFHS-4 found that half of the Muslim women did not use any hygienic methods of menstrual protection. However, within a short period of over four years, three-fourths of Muslim women used MHP. Likewise, the wealth quintile portrays that belonging to the wealthier class has a higher prevalence of using hygienic menstrual products; in NFHS-4, almost eighty-nine percent of women practiced the hygienic methods, and in NFHS-5, it reached ninety-five percent. The use of sanitary napkins as a hygienic method of menstrual protection has increased substantially and significantly, even among socio-economically deprived groups of young women. Among those who were illiterate, the use increased from 13% to 32% in the last four years. The corresponding increase among young women from SC/ST has been from 36% to 60%, and while the increase for those in the lowest wealth quintile has been from 13% to 42%. The increase in the use of sanitary napkins is also impressive among young women from rural areas (from 34% to 59%) and Muslims (from 39% to 62%).

**Table 3:** Percentage of young women aged 15–24 reporting different hygienic menstrual protection methods by selected background characteristics, India, NFHS-4 (2015–16) & NFHS-5 (2019–21).

Background characteristic	NFHS- 4				NFHS- 5			
	Locally prepared napkins	Sanitary napkins	Tampons	Using any hygienic method	Locally prepared napkins	Sanitary napkins	Tampons	Using any hygienic method
Age								
15–19	16.4	41.8	2.4	57.7	15.2	64.5	1.7	78.0
20–24	16.1	41.8	2.4	57.4	14.7	64.2	1.6	77.2
Age at menarche								
7–12	15.2	41.6	0.3	57.0	14.5	65.3	0.9	78.4
13–17	16.7	42.3	2.4	58.1	15.1	64.3	1.6	77.5
18–24	13.2	39.0	7.0	57.0	18.7	60.7	1.3	77.0
Knowledge of peak fertility								
No	16.4	40.1	2.5	56.3	15.4	62.3	1.8	76.2
Yes	17.8	47.2	2.5	63.8	15.8	68.5	1.6	81.4
Residence								
Urban	19.5	59.2	3.4	77.5	14.1	77.5	1.8	89.6
Rural	14.8	33.6	1.9	48.2	15.3	58.9	1.6	72.6
Media exposure								
No	7.5	13.4	0.7	20.9	12.6	41.7	1.2	54.1
Yes	17.9	47.1	2.7	64.4	15.5	69.7	1.8	83.1
Educational level								
No education	6.8	13.0	1.0	19.9	10.9	32.4	1.1	43.2
Primary	9.6	21.1	1.2	31.1	12.0	42.0	1.1	53.2
Secondary	17.2	43.2	2.4	60.1	15.3	64.8	1.6	78.5
Higher	22.0	65.3	3.7	85.5	15.7	79.5	1.9	92.6
Religion								
Hindu	16.5	41.3	2.4	57.2	15.1	64.0	1.6	77.3
Muslim	14.8	39.1	2.3	53.9	13.3	61.8	2.0	74.4
Others	17.5	61.7	2.3	76.8	15.4	76.1	0.9	88.4
Caste								
SC & ST	14.4	35.9	2.0	50.0	14.6	60.1	1.6	73.1
OBC	16.7	41.1	2.4	57.3	15.9	63.3	1.7	77.4
Other	17.8	50.0	2.9	67.0	13.5	70.9	1.6	82.8
Wealth quintile								
Poorest	7.6	13.2	0.9	21.1	12.0	41.7	1.4	53.6
Poor	13.5	27.2	1.7	41.3	15.3	56.8	1.7	71.1
Middle	18.4	42.3	2.5	60.4	16.3	68.0	1.6	82.1
Rich	21.3	55.9	3.2	76.2	16.3	75.6	1.9	89.0
Richest	20.1	70.8	3.8	88.8	14.8	83.1	1.7	95.1
Total	16.3	41.8	2.4	57.6	15.0	64.4	1.7	77.6

Table 4 presents the fixed and random effect models examining the factors influencing MHP among adolescents and young women aged 15–24. The null model, Model 1, and Model 2 are employed to explore clustering at the district, PSU, and household levels. In the fixed effects, Model 1 introduced contextual factors, showing significant associations between factors such as wealth, residence, caste, religion, and MHP. Higher economic status was strongly correlated with increased usage of hygienic methods in both surveys [NFHS-4 = 79.65***(59.40–92.64); NFHS-5 = 68.74***(59.68–79.17)]. Model 3 considers all background variables and reveals that age and marital status have an inverse relationship with hygienic methods, while education, economic status, and residence have a positive association. In 2015–16, women who experienced a later age at menarche were more likely to adopt MHP. However, by 2019–21 the effect of age at menarche had become statistically insignificant. A similar pattern was found for the variable “knowledge of peak fertility”, which is a proxy variable of reproductive awareness. During 2015–16, women with higher secondary or higher education were 25 times more likely to use hygienic methods by 2019–21 it increased by two units [28.70***(23.98–34.96)]. Religion and caste also influence MHP, reflecting evolving awareness and behavior patterns. In the random effect, the null model, which serves as the baseline, indicates that household-level clustering explains a substantial portion of the overall variation in MHP in both the NFHS rounds. After adding contextual factors, the intra class correlation (ICC) values exhibited a significant decline at all levels, from the null Model to Model 2, in both rounds. Although the ICC values changed in Model 1 compared to the null model, the household-level factors remained a significant source of clustering. Model 2 considers all background characteristics of women, providing a more comprehensive understanding of the factors influencing MHP. The household-level ICC remained significantly high at 78.49% in NFHS-4 and 78.87% in NFHS-5, reaffirming the role of household-level clustering in explaining the variation in MHP. Overall, the addition of contextual and background factors in Model 1 and Model 2 does not significantly alter the importance of household-level clustering. This suggests that even when accounting for various individual and contextual factors, household-level dynamics and shared characteristics still play a crucial role in shaping MHP among adolescents and young women in India.

**Table 4:** Random effect and fixed effect model showing key drivers of hygienic menstrual practice among adolescents and young women aged 15–24 according to background characteristics, India, NFHS-4 (2015–16) & NFHS-5 (2019–21).

Background characteristic	NFHS-4			NFHS-5		
	Null model	Model 1	Mode l2	null model	Model 1	Model 2
	OR (95% CI)	OR (95% CI)	OR (95% CI)	OR (95% CI)	OR (95% CI)	OR (95% CI)
Age						
15–19 ®						
20–24			0.71*** (0.65–0.76)			0.70*** (0.64–0.76)
Age at Menarche						
7–12®						
13–17			1.23*** (1.11–1.36)			1.05 (0.96–1.15)
18–24			1.73** (1.15–2.60)			1.27 (0.72–1.74)
Knowledge of peak fertility						
NO®						
Yes			1.43*** (1.26–1.63)			1.36 (1.21–1.53)
Current marital status						
Not married ®						
Married			0.74*** (0.67–0.82)			0.81*** (0.74–0.89)
Media exposure						
No®						
Yes			2.88*** (2.53–3.27)			2.76*** (2.43–3.12)
Education level						
No education ®						
Primary			1.68*** (1.47–1.93)			1.61***(1.38–1.88)
Secondary			6.08*** (5.05–7.33)			5.80*** (4.70–7.13)
Higher secondary & above			25.24*** (18.61–34.24)			28.70*** (23.98–34.96)
Wealth quintile						
Poorest ®						
Poor		4.25*** (3.97–5.53)	3.25*** (2.78–3.78)		3.72*** (3.48–3.97)	2.72*** (2.37–3.12)
Middle		13.46*** (12.41–14.59)	8.60*** (6.61–11.17)		9.71*** (8.92–10.56)	5.85*** (4.72–7.24)
Rich		41.84*** (37.87–46.22)	24.89*** (17.26–35.87)		22.62*** (20.36–25.12)	11.92*** (8.99–15.80)
Richest		169.65*** (149.40–192.64)	88.12*** (54.62–142.17)		68.74*** (59.68–79.17)	34.31*** (23.11–50.95)
Residence						
Rural ®						
Urban		1.96*** (1.83–2.10)	2.15*** (1.89–2.45)		1.84*** (1.68–2.02)	2.00*** (1.75–2.29)
Religion						
Hindu ®						
Muslim		0.49*** (0.45–0.53)	0.69*** (0.60–0.81)		0.58*** (0.53–0.63)	0.78*** (0.68–0.90)
Other		2.23*** (1.91–2.60)	1.91*** (1.56–2.33)		1.45*** (1.26–1.65)	1.68*** (1.33–2.13)
Caste/tribe						
SC/ST®						
Other backward class		1.2*** (1.13–1.26)	1.10** (1.02–1.18)		1.26*** (1.19–1.33)	1.15*** (1.06–1.25)
Non-SC/ST & non OBC		2.01*** (1.88–2.14)	1.81*** (1.62–2.03)		1.60*** (1.48–1.73)	1.43*** (1.27–1.63)
Constant		0.14*** (0.13–0.17)	0.02*** (0.01–0.03)		2.7*** (2.4–3.1)	0.47*** (0.38–0.59)
Var (district)	5.91 (5.20–6.70)	2.09 (1.85–2.37)	2.17 (1.70–2.77)	4.8 (4.23–5.40)	2.5 (2.19–2.82)	2.36 (1.91–2.91)
Var (PSU)	3.88 (3.70–4.07)	2.03 (1.92–2.14)	2.29 (1.85–2.83)	3.5 (3.34–3.72)	2.4 (2.27–2.56)	2.40 (1.95–2.94)
Var (HHs)	6.39 (6.08–6.71)	5.28 (4.99–5.57)	7.54 (5.75–9.89)	6.7 (6.36–7.17)	5.9 (5.59–6.32)	7.52 (5.67–9.97)
ICC (district) (%)	30.33 (27.87–32.90)	16.50 (14.90–18.22)	14.21 (12.54–16.06)	26.1 (23.8–28.4)	17.6 (15.9–19.4)	15.18 (13.64–16.85)
ICC (PSU) (%)	50.28 (48.45–52.12)	32.47 (31.04–33.93)	29.17 (27.34–31.08)	45.3 (43.4–47.1)	34.8 (33.1–36.1)	30.58 (28.92–32.29)
ICC (HHs) (%)	83.10 (82.21–83.95)	74.06 (73.02–75.09)	78.49 (74.06–82.34)	82.1 (81.1–82.9)	76.7 (75.6–77.7)	78.87 (74.51–82.65)

*p < 0.05, **p < 0.01, ***p < 0.001.

Table 5 reveals the disparity in the adoption of MHP among women aged 19–24 between 2015 and 2019 using Fairlie decomposition analysis. The study found that media exposure (118.68%) was the most significant positive contributor, underscoring the powerful role of awareness campaigns in minimizing disparities. Improvements in educational attainment have played a substantial role in reducing disparities by reducing the gap by thirty-eight percent in MHP from 2015–16 to 2019–21, suggesting better education effectively translates into better MHP. Similarly, the wealth quintile (16.28%) and place of residence (4.62%) contributed to reducing inequalities. Whereas, the effect of caste became insignificant.

**Table 5:** Fairlie decomposition of hygienic menstrual practice among adolescents and young women aged 15–24, India, NFHS-4 (2015–16) & NFHS-5 (2019–21).

Background characteristics	Coefficient	*p*-value	95% CI		Percent contribution
			Lower limit	Upper limit	
Age	−0.00012	0.008	−0.00020	−0.00003	−0.29
Education level	0.01473	0.000	−0.01520	−0.01427	−37.59
Marital status	−0.00151	0.000	−0.00183	−0.00119	−3.85
Media exposure	0.04651	0.000	0.04416	0.04886	118.68
Wealth quintile	0.00638	0.000	0.00621	0.00655	16.28
Place of residence	0.00181	0.000	0.00160	0.00202	4.62
Caste	0.00007	0.414	−0.00010	0.00024	0.18
Religion	0.00081	0.000	0.00061	0.00100	2.06

## 4 Discussion

In India, almost 355 million women and girls are at the age of menstruation. Menstruation is accompanied by both physiological and psychological changes. Stigma, customs, culture, and rituals significantly impact menstruators and limit their access to hygienic menstrual products. Most women face stigma and struggle to access hygienic menstrual products (31). Several studies have reported that approximately sixty-six percent of women aged 15–24 use cloths during menstruation, and 17% use locally prepared napkins. These results were persistent in the Indian scenario (17, 32, 33).

This study presents a micro and macro perspective of factors affecting MHP at the country, state, district, and individual levels. This study found that hygienic menstrual absorbents are less common in rural areas than in urban areas. However, the widespread use of hygienic menstrual methods has significantly improved from 2016 to 2021. These differentials portray equity in the use of hygienic methods of menstrual protection over time by urban‒rural place of residence, educational attainment of women, and socio-economic deprivation. The findings of the study indicate the changing role of cultural ecology in improving menstrual hygiene practices across India's diverse states. Interstate variability in MHP is another critical challenge for ensuring universalization in the country. States such as Madhya Pradesh (53%), Bihar (57%), Meghalaya (59%), and Chhattisgarh (61%) had a relatively lower prevalence of MHP among young rural women than other states. These findings are in line with several other studies reflecting the fact that awareness, affordability, and accessibility of the hygienic methods of menstrual products are the primary causes for not using hygienic methods (14, 34–38).

Furthermore, the study presents a geospatial map of India to show temporal changes in MHP in the last four years. From 2015–16 to 2019–21, the use of MHP increased, which exhibits effective planning of government schemes “Menstrual Hygiene Scheme (MHS)” and “SUVIDHA”. The Ministry of Health and Family Welfare launched MHS in 2011 to promote menstrual hygiene among adolescent girls aged 10–19 years in rural areas as a part of Adolescent Reproductive Sexual Health (ARSH) in RCH (39), and SUVIDHA was launched in 2018 under the Pradhan Mantri Bhartiya Janaushadi Pariyojna to provide the 100% Oxo-bridgeable Sanitary Napkin available for Rs. 2.50 per napkin over 3200 Janaushadi Kendra across India (40).

To better understand the spatial variation in MHP and to examine temporal changes, the LISA map was used to identify the spatial dependency and clustering with some significant predictors that emerged during micro-level models. This study calculated the Moran's I indices and presented a bivariate scatter plot. The findings revealed that districts with maximum exposure to mass media also had a higher rate of MHP. This implies that the extent of awareness regarding menstrual hygiene is crucial to escalating the use of hygienic menstrual methods. Other significant covariates leading to spatial variations in MHP were educational attainment, improved sanitation, and economic prosperity. These findings are similar to several other studies that have explained that the individual's education is a more significant factor in increasing the practice of hygienic menstrual methods (16, 19). Many studies have interchangeably used educational attainment and media exposure (20, 33, 38).

The spatial variation in the use of hygienic methods of menstrual protection was not uniform between 2016 and 2021. The geospatial interdependency of the outcome variable by the selected set of predictor variables portrays that educational attainment, exposure to mass media, economic wellbeing, and caste have a statistically significant impact on the use of menstrual hygiene methods in both periods. Nevertheless, the degree of association weakened over time. However, there was no spatial variability in the degree of association between urban places of residence and the use of hygienic methods of menstrual protection. This may be primarily due to two reasons. First, young women in urban areas may have better awareness, accessibility, and affordability to procure hygienic methods of menstrual protection. Second, various government-sponsored schemes, such as RMNCH+A, have been implemented for a decade, under which many self-help groups and ASHA workers are taking care of menstrual hygiene management to address the overall reproductive and sexual health of adolescents and young women in the country. However, the intensity of such programs among rural residents varies across states, and hence needs to be prioritized and upscaled across the country (41).

Another finding of this study revealed that sanitary napkins are more commonly used to prevent blood stains during menstruation than any other hygienic menstrual product among adolescents and young women aged 15–24 in India, which cuts across the caste and class of women. This may have been the outcome of government-sponsored initiatives and programs to enhance access to low-cost biodegradable sanitary napkins within a larger framework to promote adolescent reproductive health in the country. This plausible reason behind the increased utilization of sanitary napkins is also supported by the fact that there was no change in the proportion of young women who reported using locally prepared sanitary napkins in India from 2016 to 2021. It is worth mentioning here that the information on the critical barriers in procuring the hygienic methods of menstrual protection by those using cloths, cotton wool, toilet paper, and underwear only would have helped strengthen the narratives of change and design suitable programs to eliminate those obstacles in procuring hygienic methods to collect or absorb menstrual blood (21, 32, 37).

The multilevel analysis at the district, PSU, and household levels shows that the maximum variation in practising the hygienic method is present at the household level, followed by the PSU and district levels, implying that the use of MHP is primarily affected by household-level factors, which may be governed by a large set of socio-cultural and developmental variables influencing the environment and ecology of their living. This leads to contemplating the customs, stigma, and perspectives on hygienic menstruation. Khanna et al. found that almost seventy percent of girls were confident that menstruation was unnatural. Four out of five girls believed attending any religious rituals during menstruation was unacceptable. However, some parts of India celebrate ceremonies at menarche, such as Ritu Kala Sanskara, Manjal Neerattu Vizha, and “Xoru Biya” (2, 42, 43). These findings are adequately supported by the changing propensity of adolescents and young women aged 15–24 to practice hygienic menstrual methods from 2016 to 2021 after adjusting for other predictors included in the model. Women from economically well-off households have the highest propensity to adopt MHP, followed by education and those who live in urban areas. Scheduled caste and scheduled tribe women are relatively less likely to use hygienic methods of menstrual protection and hence are the most vulnerable group to suffer from various forms of reproductive morbidities in Indian society, a similar finding to those reported by Vishwakarma (17). These findings pose dual challenges of enhancing awareness to address stigma and discrimination associated with menstruation, especially among those who are not or have low education status, and ensuring hygienic methods of menstrual protection, especially among those who are socially deprived and economically marginalized in rural areas. Random components in the three-stage logistic regression highlighted the maximum contribution of household and PSU level factors in explaining the residuals, focusing on unexplained variation in the use of MHP among young women in India. Therefore, universalizing hygienic methods of menstrual protection as a key strategy to protect young women from various forms of reproductive morbidities and improve their quality of life should be designed and implemented at the community level, preferably using a peer-based approach.

The Fairlie decomposition analysis underscores persistent disparities in the adoption of MHP among young women despite improvements in education and media exposure. While education has narrowed the gap, the disparity remains, suggesting that factors beyond formal schooling, such as cultural norms and access to menstrual products, continue to limit progress. Media exposure significantly reduced disparities, but the reliance on awareness campaigns raises concerns about sustainability without ongoing interventions. The diminishing role of caste suggests a shift in traditional social barriers, yet disparities linked to wealth and residence persist, indicating broader socio-economic challenges. The persistence of these disparities requires a multifaceted approach to address the systemic inequalities.

Although expanding access to low-cost sanitary pads has played a critical role in improving menstrual hygiene across India, it has also created new challenges related to environmental sustainability and public health. Most disposable pads contain up to 90% plastic and synthetic polymers, which are non-biodegradable and generate significant waste (44). Improper disposal, such as open dumping or incineration can lead to soil and water pollution, as well as harmful emissions including dioxins and furans that threaten both environmental and human health (45). In low-resource settings, inadequate waste management infrastructure exacerbates these risks, making it essential to reconsider the environmental costs of menstrual health interventions. Emerging sustainable alternatives such as bio-degradable pads offer promising solutions. A recent feasibility study conducted in Karnataka evaluated banana fiber-based sanitary pads and found them to be acceptable, compostable, and effective in meeting users’ needs (46). Promoting such eco-friendly options, along with menstrual cups and reusable cloth pads, could reduce the environmental burden, while safeguarding women's health.

This study imitates the MHP in Indian settings at every level between the two periods. From 2016 to 2021, there has been a significant and profound change in MHP among adolescents and young women aged 15–24 despite existing cultural and ecological taboos associated with menstruation. In every state, MHP have increased. In these four years, a substantial proportion of young women started to use sanitary napkins more than other hygienic methods of menstrual protection. MHP in India are highly influenced by geospatial attributes, culture, and tradition compared to other factors. At the district level, geospatial analysis shows a close spatial clustering in MHP, which is majorly affected by women's education level, belonging to the economically well-off household, exposure to mass media, and improved sanitation facilities.

## 5 Conclusion

This study provides a comprehensive analysis of MHP among young women in India, highlighting significant improvements from 2016 to 2021 while also revealing persistent disparities. Study shows increased adoption of MHP across India, with sanitary napkins becoming the most common method with a persistent disparity among socially and economically marginalized groups, with rural areas lagging behind. Another finding showed significant interstate variability in the prevalence of MHP, with some states showing lower adoption rates. Further, spatial analysis revealed clustering of MHP, influenced by factors such as education, media exposure, economic status, and household-level factors contributing most to the variation in MHP, followed by community- and district-level factors. Education, economic status, and urban residence have emerged as strong predictors of the adoption of MHP.

## Data Availability

Publicly available datasets were analyzed in this study. This data can be found here: https://dhsprogram.com/data/.
