# Supplementary material for: Exploring the temporal shift in menstrual hygiene practices among young women across India: a micro and macro perspectives
Source: Front Reprod Health. 2025 Jul 30;7:1532178. doi: 10.3389/frph.2025.1532178 (PMC12343603; doi:10.3389/frph.2025.1532178)
Supplement: Supplementary file 1 [file Table1.docx]

Table: Diagnostic test results of the spatial regression model, NFHS 4 and NFHS 5, India

|  | NFHS-4 | NFHS-5 |
| --- | --- | --- |
| Lagrange Multiplier (lag) | 0.65 | 0.59 |
| Lagrange Multiplier (error) | 0.81 | 0.76 |
| Akaike Information Criterion (AIC) | 4516.22 | 4795.2 |
