# Supplementary material for: Exploring the temporal shift in menstrual hygiene practices among young women across India: a micro and macro perspectives
Source: Front Reprod Health. 2025 Jul 30;7:1532178. doi: 10.3389/frph.2025.1532178 (PMC12343603; doi:10.3389/frph.2025.1532178)
Supplement: Supplementary file 2 [file Supplementaryfile1.docx]

Supplementary Material

# Supplementary Figures and Tables

**Figure 4(A):** Bivariate significance map of hygienic menstrual practice among adolescents and young women aged 15-24 with their education India, 2015-16 (NFHS 4)

**Figure 4(B):** Bivariate significance map of hygienic menstrual practice among adolescents and young women aged 15-24 with their wealth status. India, 2015-16 (NFHS 4)

**Figure 4(C):** Bivariate significance map of hygienic menstrual practice among adolescents and young women aged 15-24 with improved sanitation facility, India, 2015-16 (NFHS 4)

**Figure 4(D):** Bivariate significance map of hygienic menstrual practice among adolescents and young women aged 15-24 with media exposure, India, 2015-16 (NFHS 4)

**Figure 4(E):** Bivariate significance map of hygienic menstrual practice among adolescents and young women aged 15-24 with their education India, 2019-21 (NFHS 5)

**Figure 4(F):** Bivariate significance map of hygienic menstrual practice among adolescents and young women aged 15-24 with their wealth status. India, 2019-21 (NFHS 5)

**Figure 4(G):** Bivariate significance map of hygienic menstrual practice among adolescents and young women aged 15-24 with improved sanitation facility, India, 2019-21 (NFHS 5)

**Figure 4(H):** Bivariate significance map of hygienic menstrual practice among adolescents and young women aged 15-24 with media exposure, India, 2019-21 (NFHS 5)

**Figure 5(A)** Local Moran’s I of education, India, 2019-21 (NFHS 5)

**Figure 5(B)** Local Moran’s I of wealth Status, India, 2019-21 (NFHS 5)

**Figure 5(C)** Local Moran’s I of sanitation facility, India, 2019-21 (NFHS 5)

**Figure 5(D)** Local Moran’s I of media exposure, India, 2019-21 (NFHS 5)

**Figure 6(A)** Randomised histogram of Local Moran’s I of education with 999 permutations, India, 2019-21 (NFHS 5)

**Figure 6(B)** Randomised histogram of Local Moran’s I of wealth status with 999 permutations, India, 2019-21 (NFHS 5)

**Figure 6(C)** Randomised histogram of Local Moran’s I of sanitation facility with 999 permutations, India, 2019-21 (NFHS 5)

**Figure 6(D)** Randomised histogram of Local Moran’s I of media exposure with 999 permutations, India, 2019-21 (NFHS 5)
