# Supplementary figures and images for: Exploring the temporal shift in menstrual hygiene practices among young women across India: a micro and macro perspectives
Source: Front Reprod Health. 2025 Jul 30;7:1532178. doi: 10.3389/frph.2025.1532178 (PMC12343603; doi:10.3389/frph.2025.1532178)

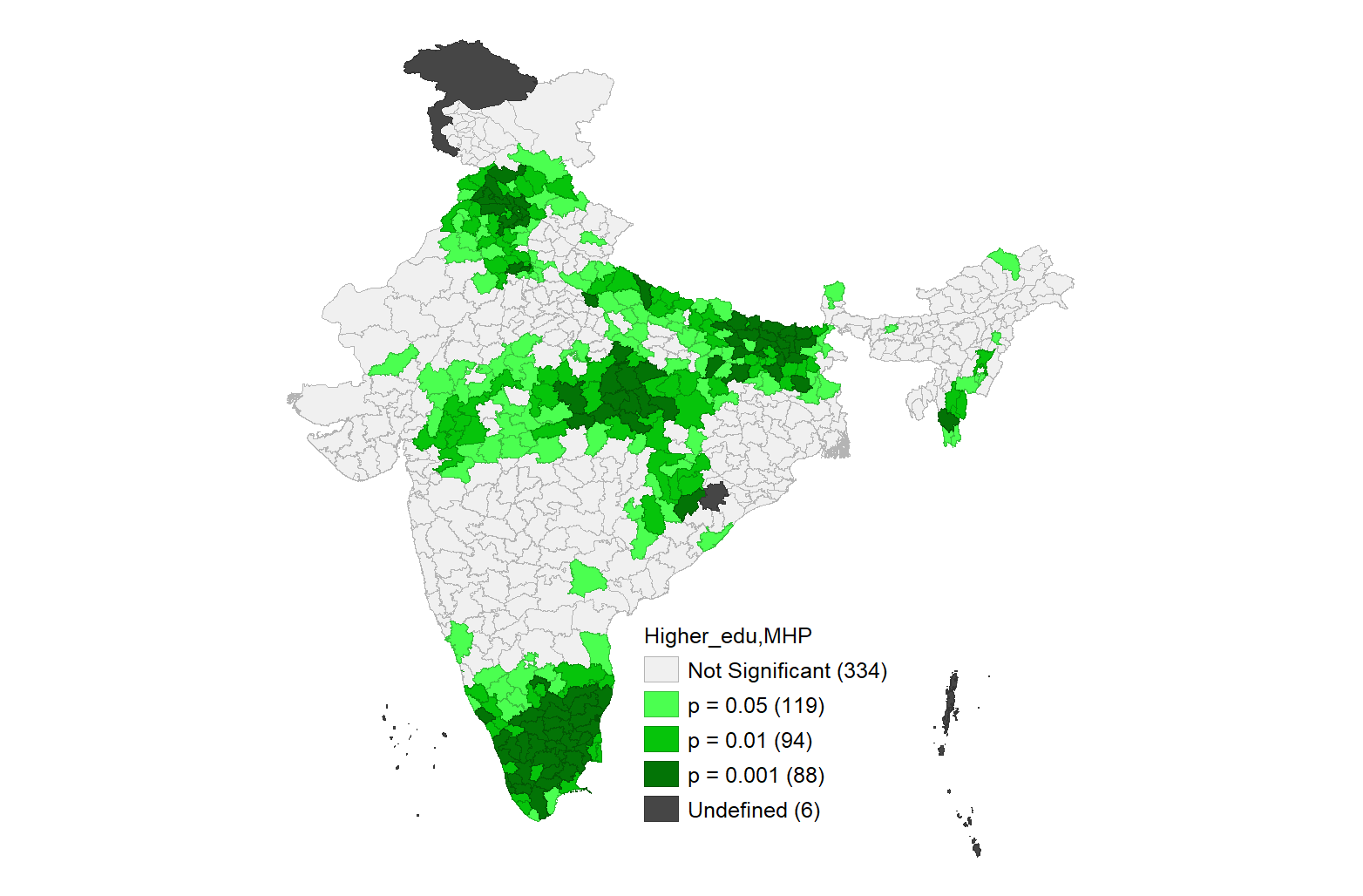

Supplement: Supplementary file 3 [file Datasheet1.zip › 4(A).tiff]

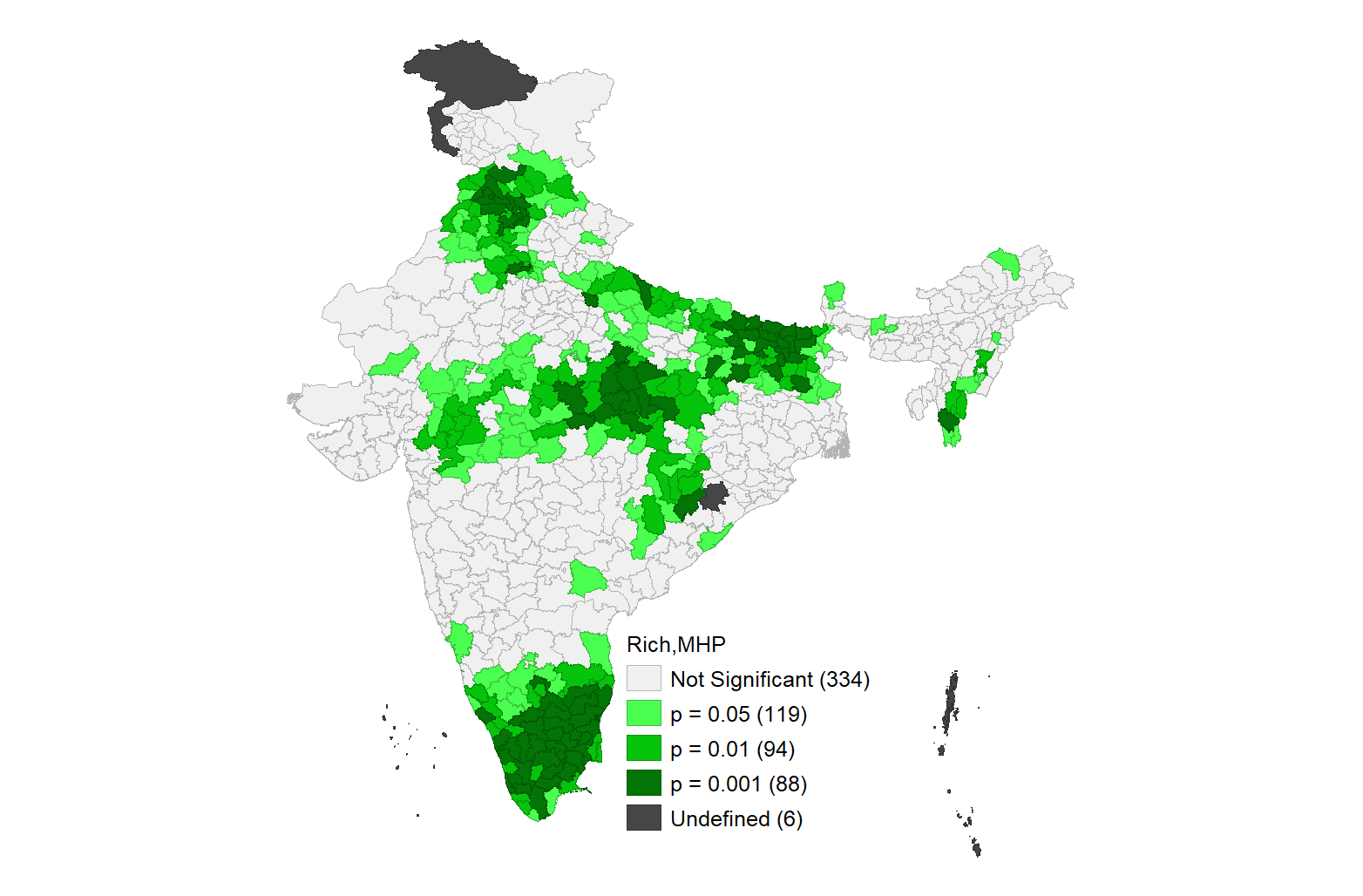

Supplement: Supplementary file 3 [file Datasheet1.zip › 4(B).tiff]

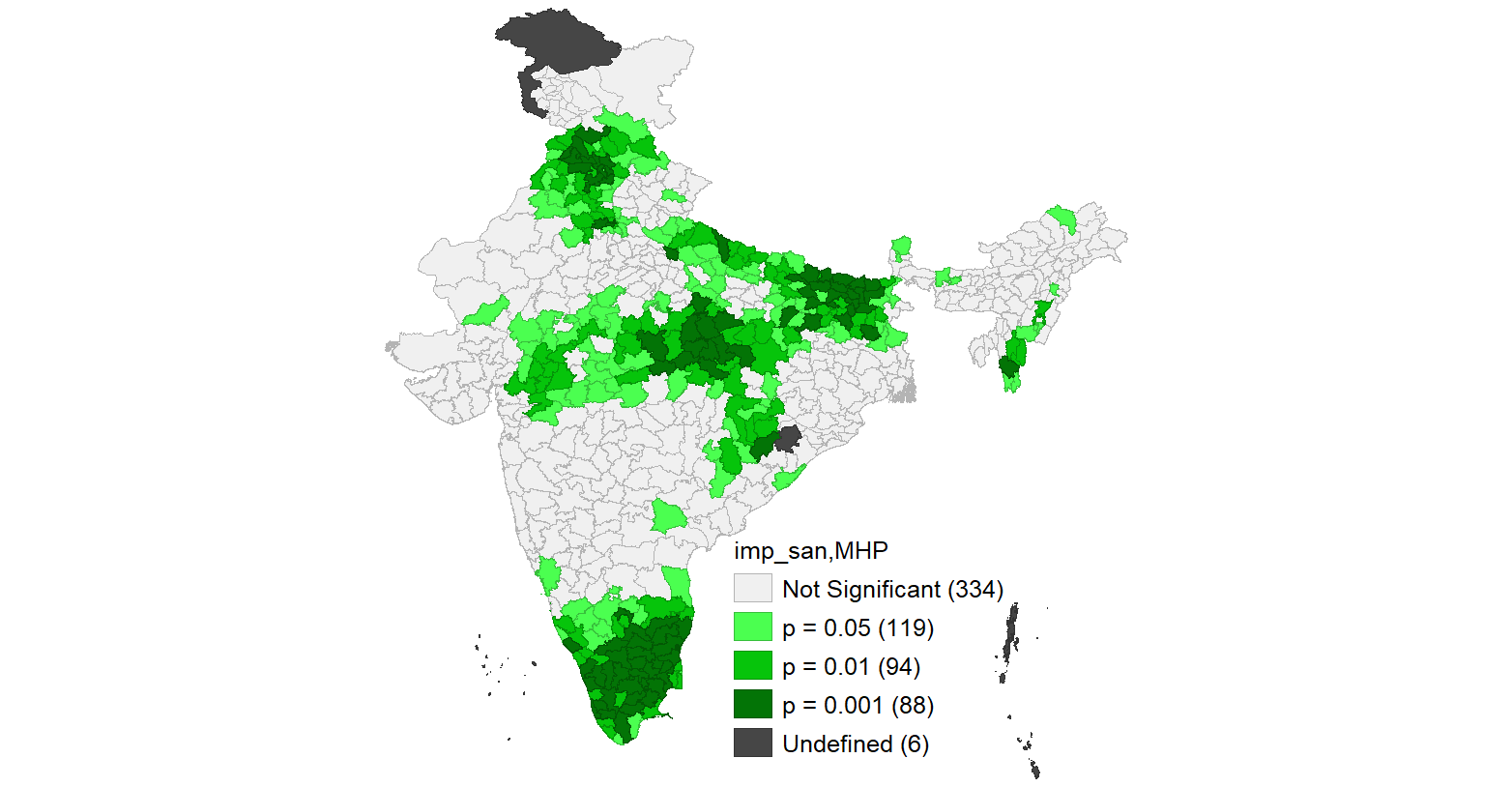

Supplement: Supplementary file 3 [file Datasheet1.zip › 4(C).tiff]

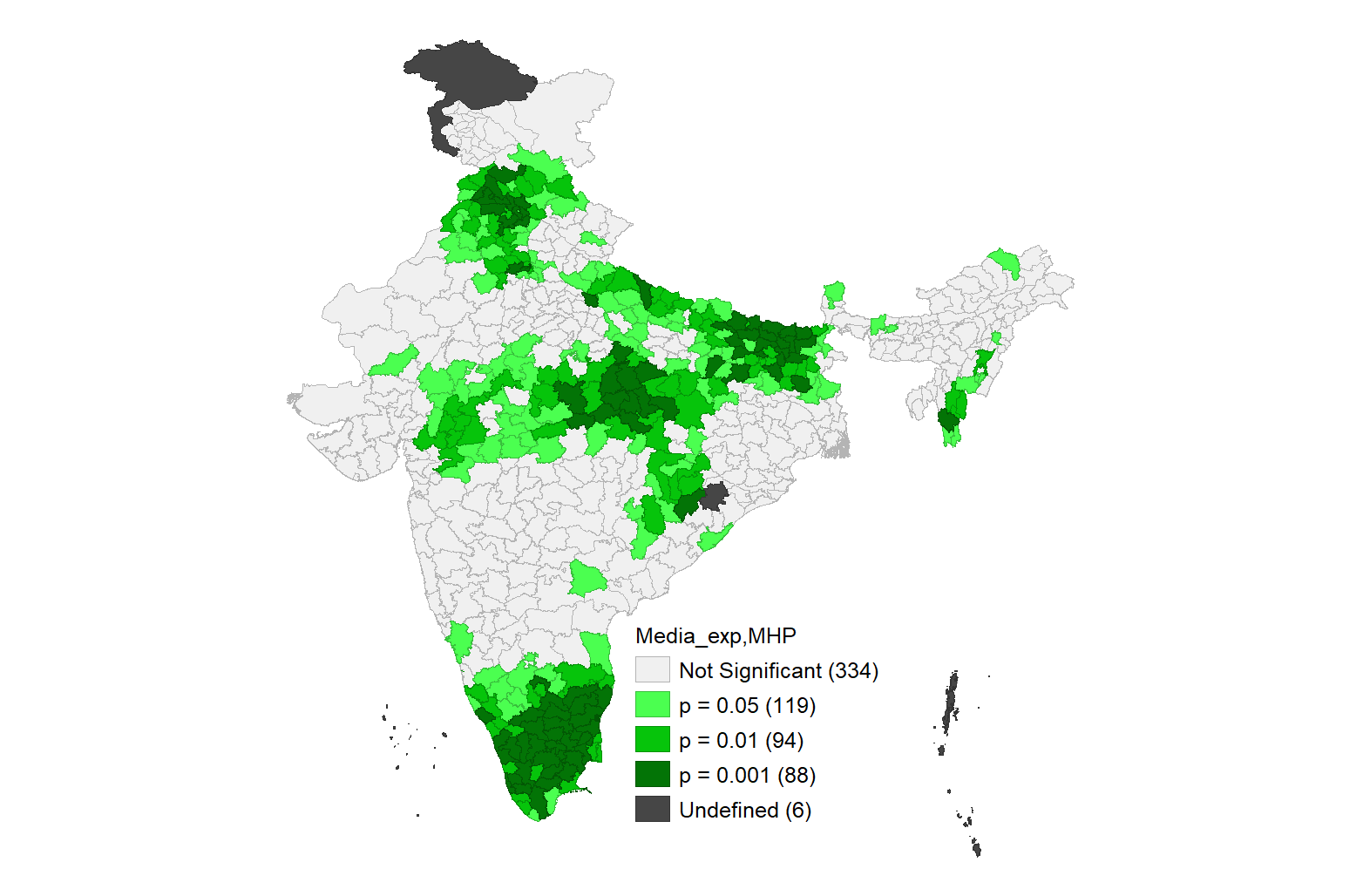

Supplement: Supplementary file 3 [file Datasheet1.zip › 4(D).tiff]

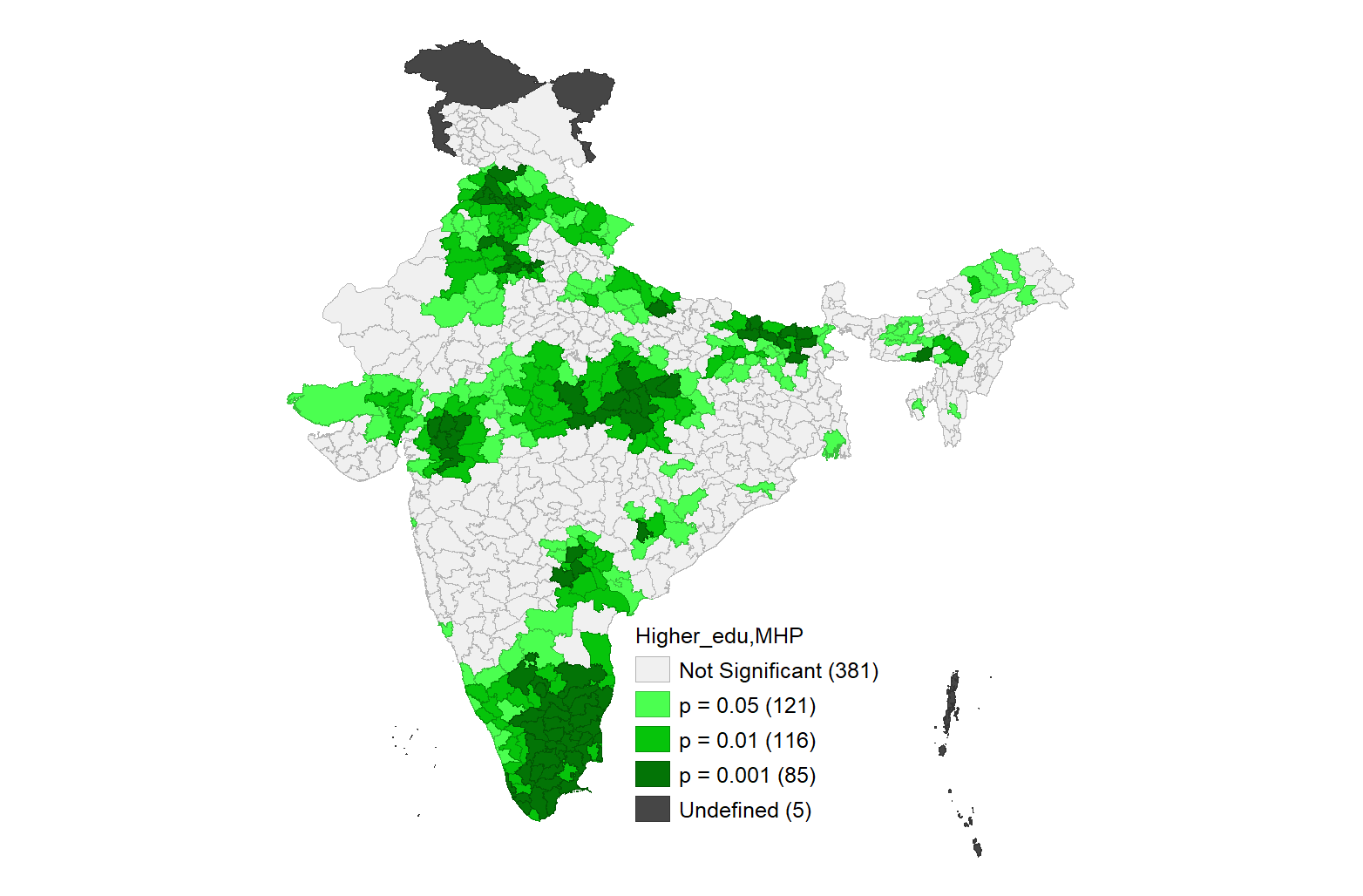

Supplement: Supplementary file 3 [file Datasheet1.zip › 4(E).tiff]

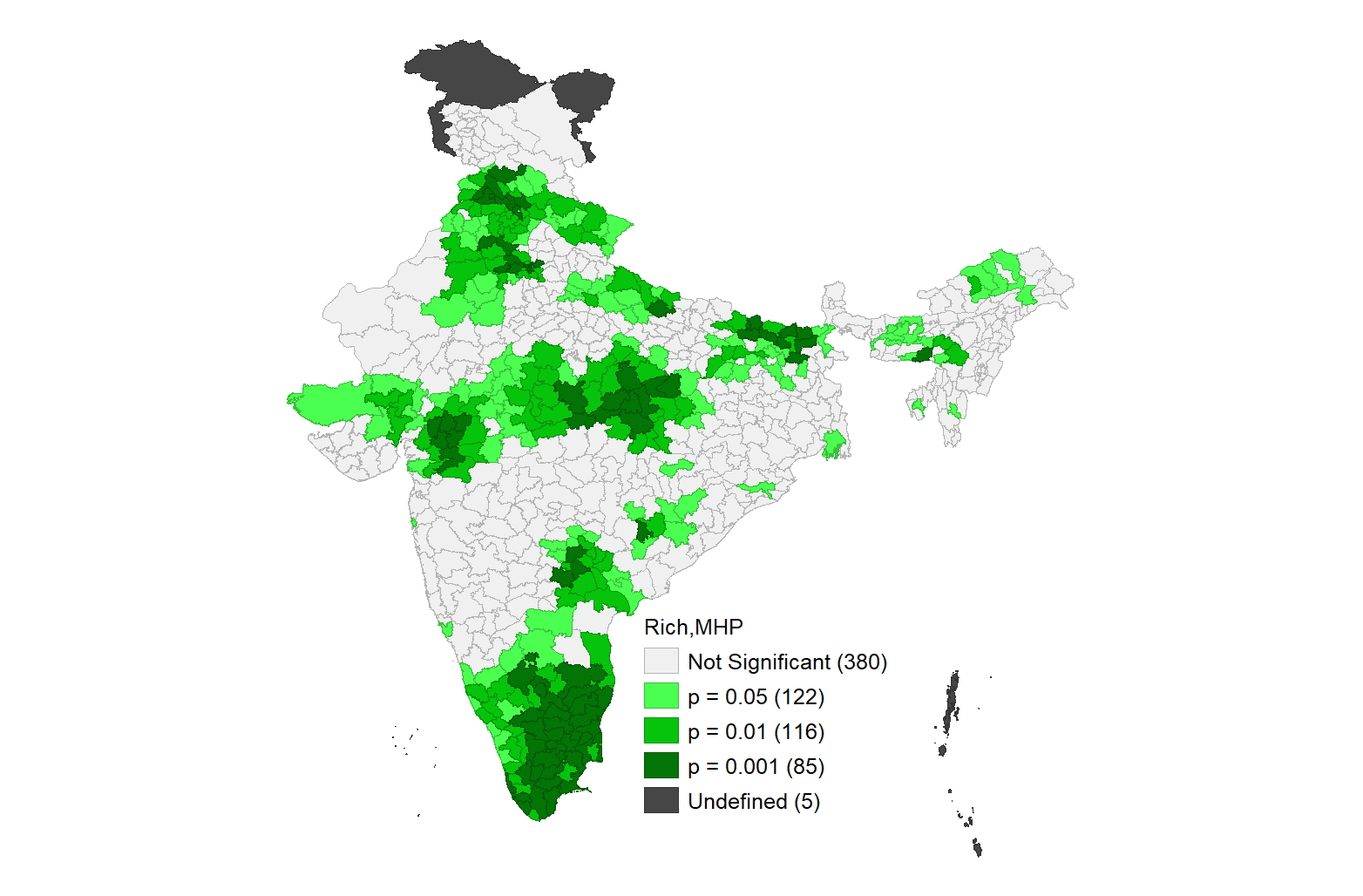

Supplement: Supplementary file 3 [file Datasheet1.zip › 4(F).tiff]

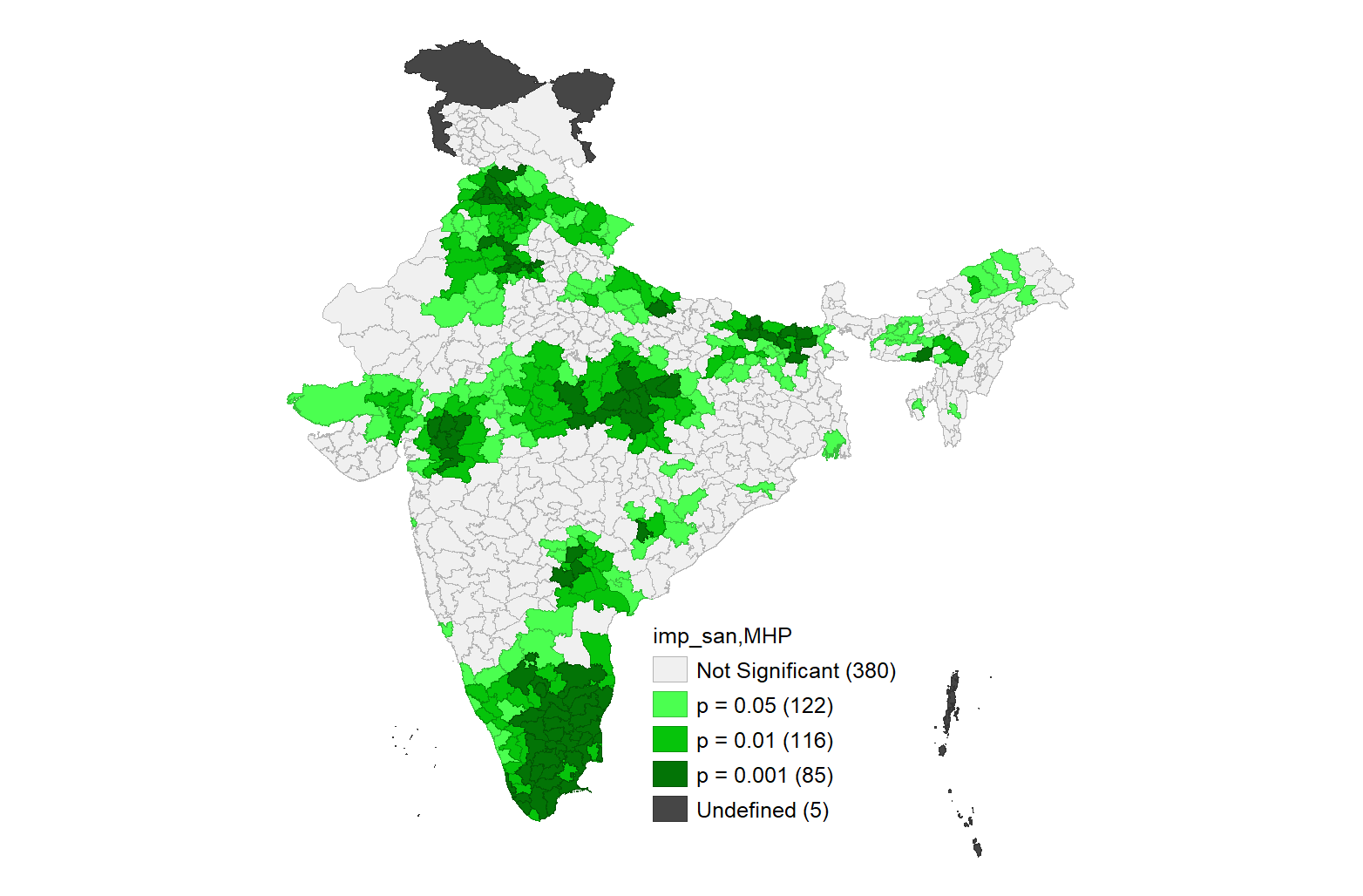

Supplement: Supplementary file 3 [file Datasheet1.zip › 4(G).tiff]

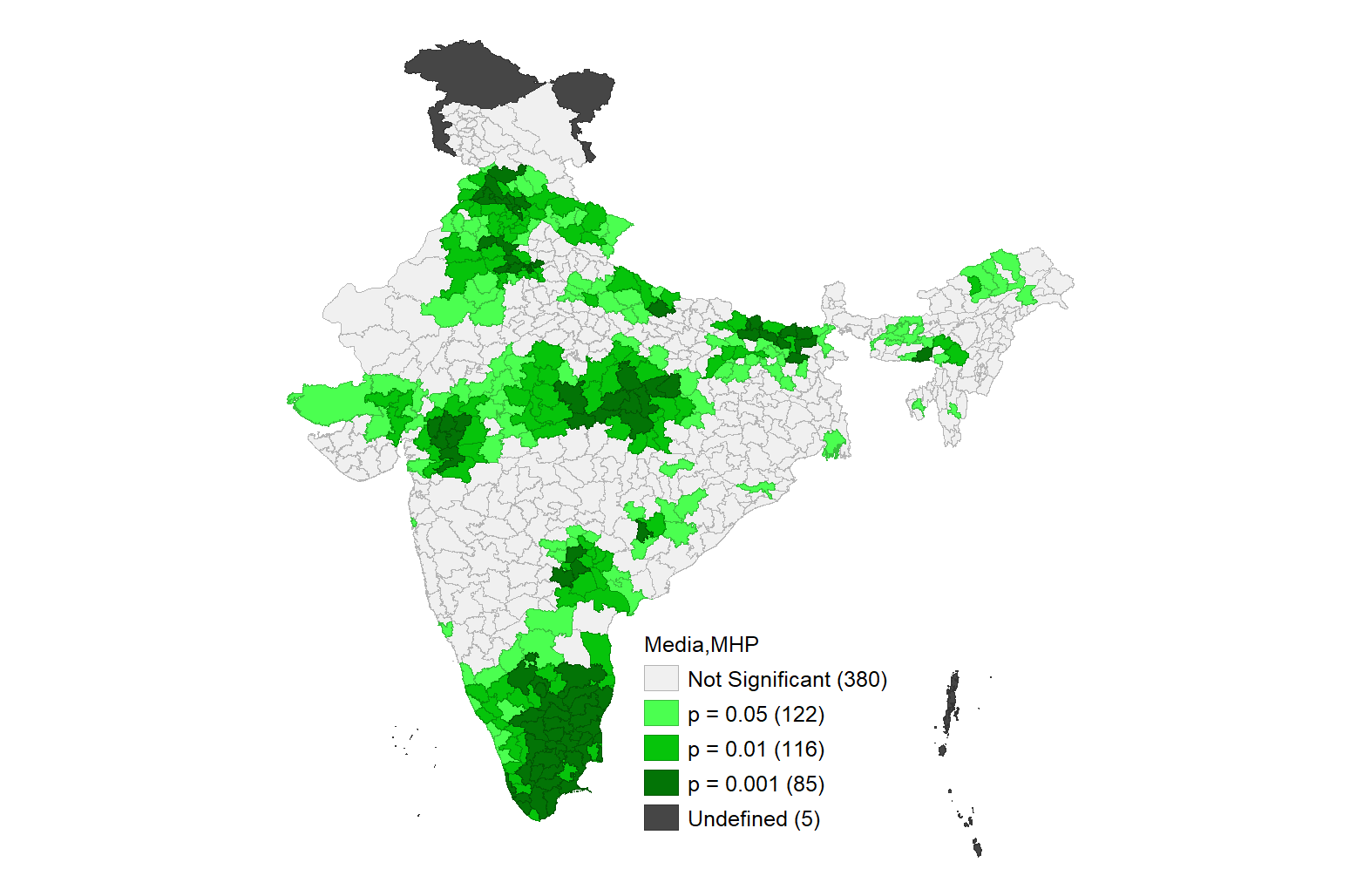

Supplement: Supplementary file 3 [file Datasheet1.zip › 4(H).tiff]

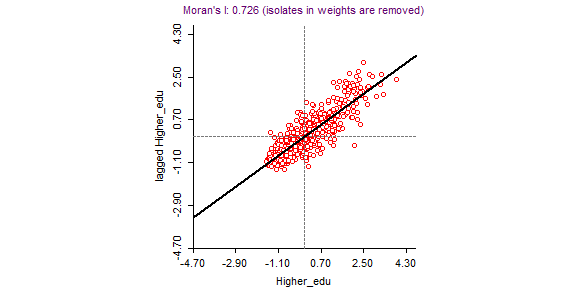

Supplement: Supplementary file 3 [file Datasheet1.zip › 5(A).png]

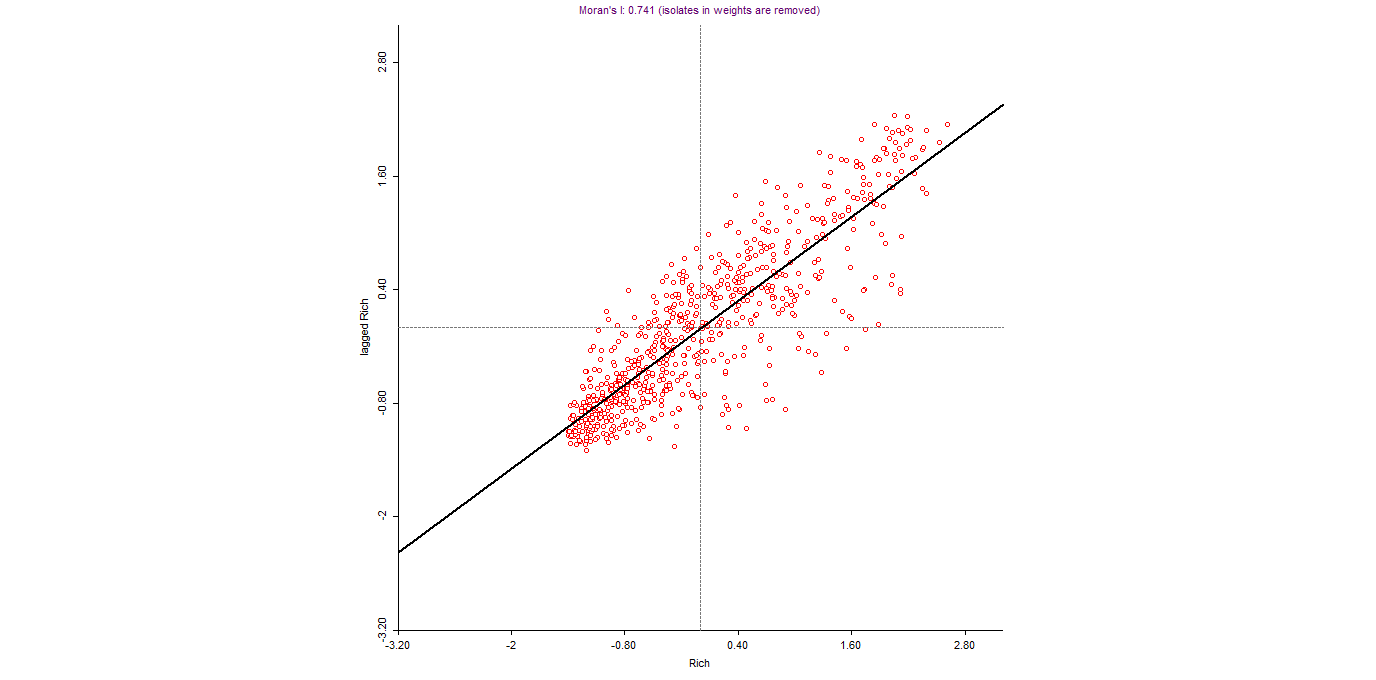

Supplement: Supplementary file 3 [file Datasheet1.zip › 5(B).png]

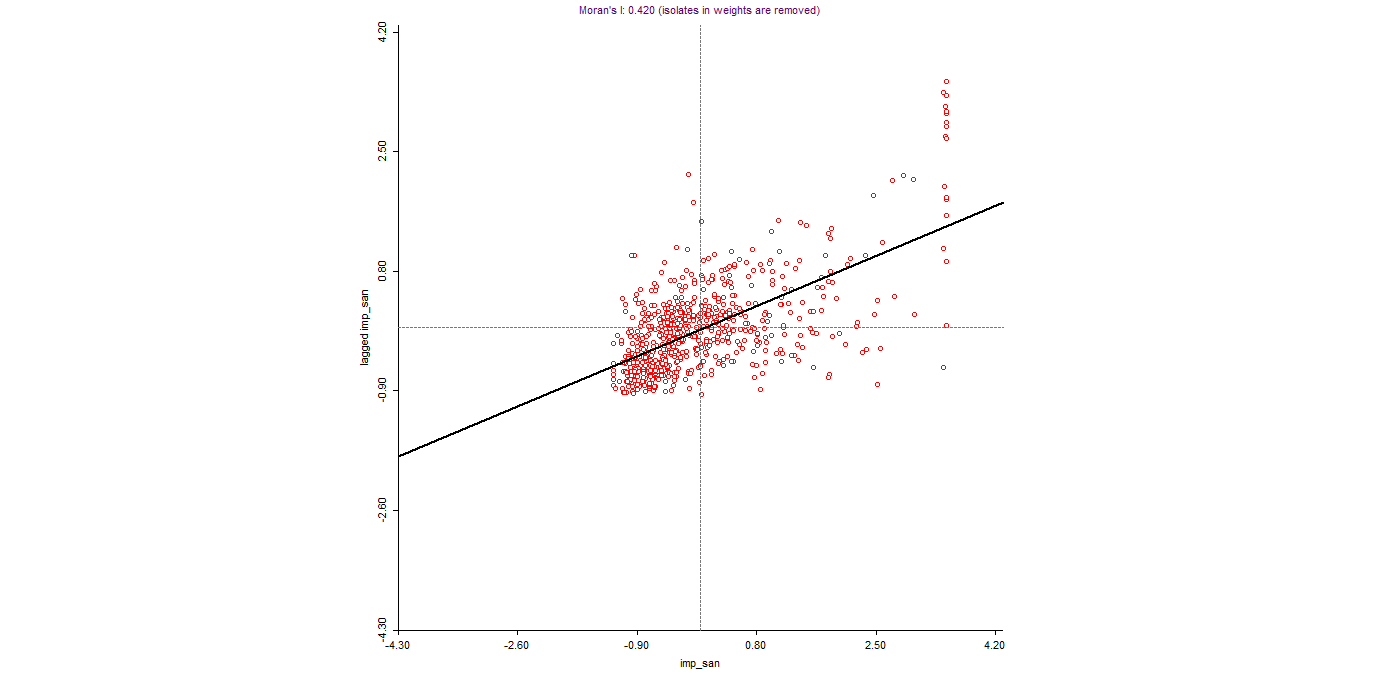

Supplement: Supplementary file 3 [file Datasheet1.zip › 5(C).png]

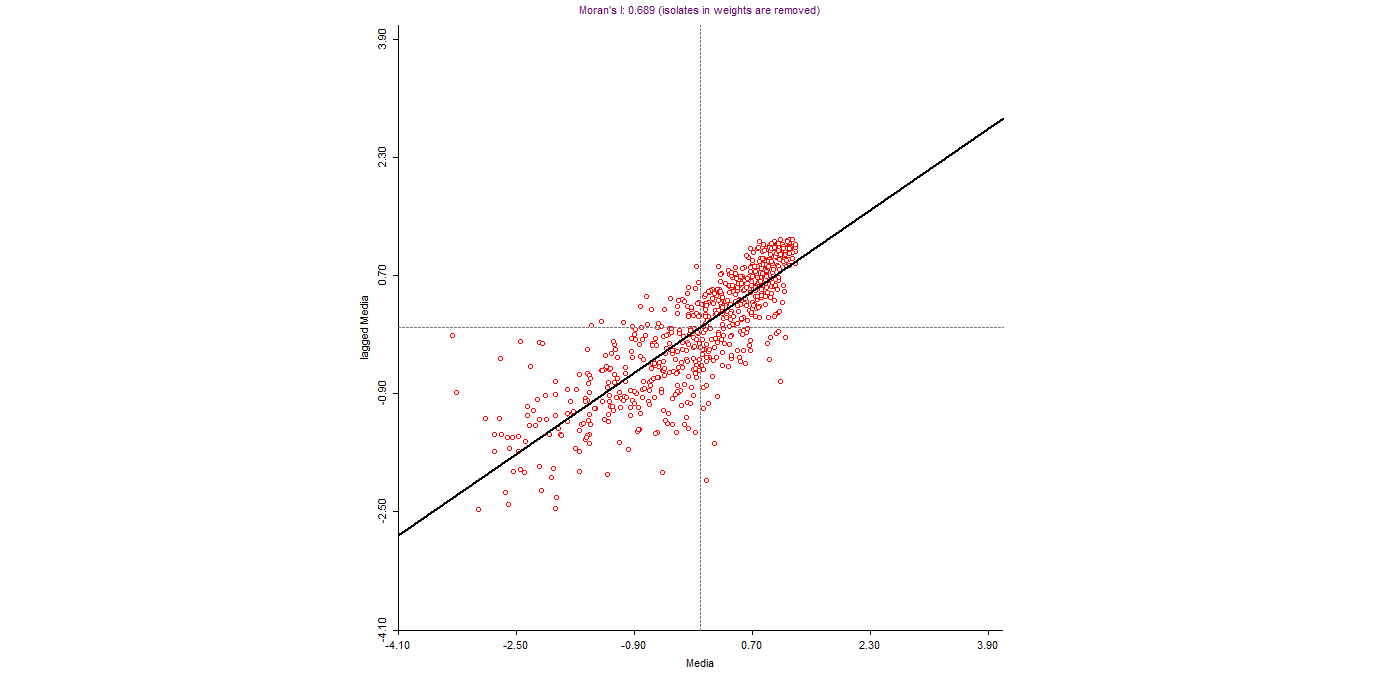

Supplement: Supplementary file 3 [file Datasheet1.zip › 5(D).png]

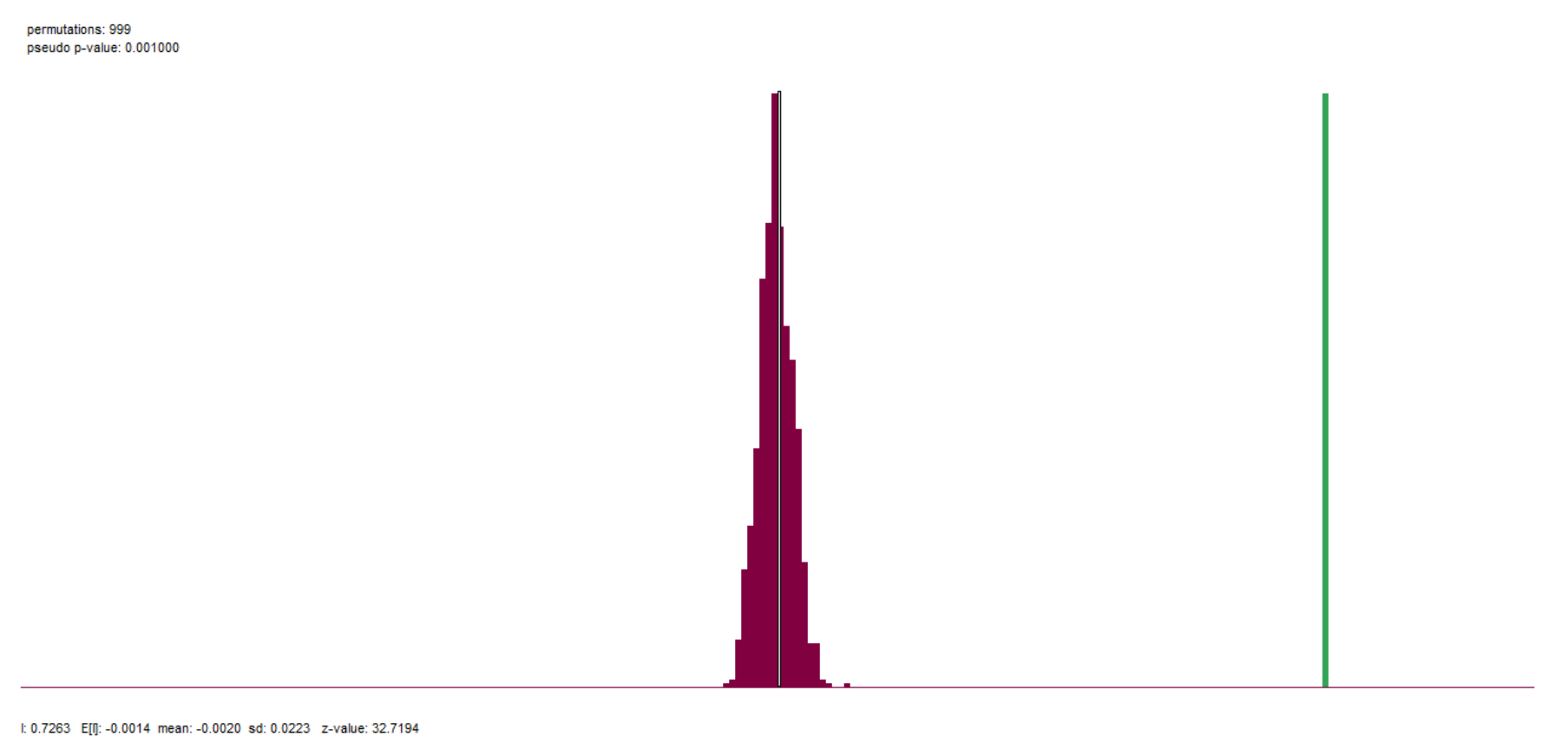

Supplement: Supplementary file 3 [file Datasheet1.zip › 6(A).png]

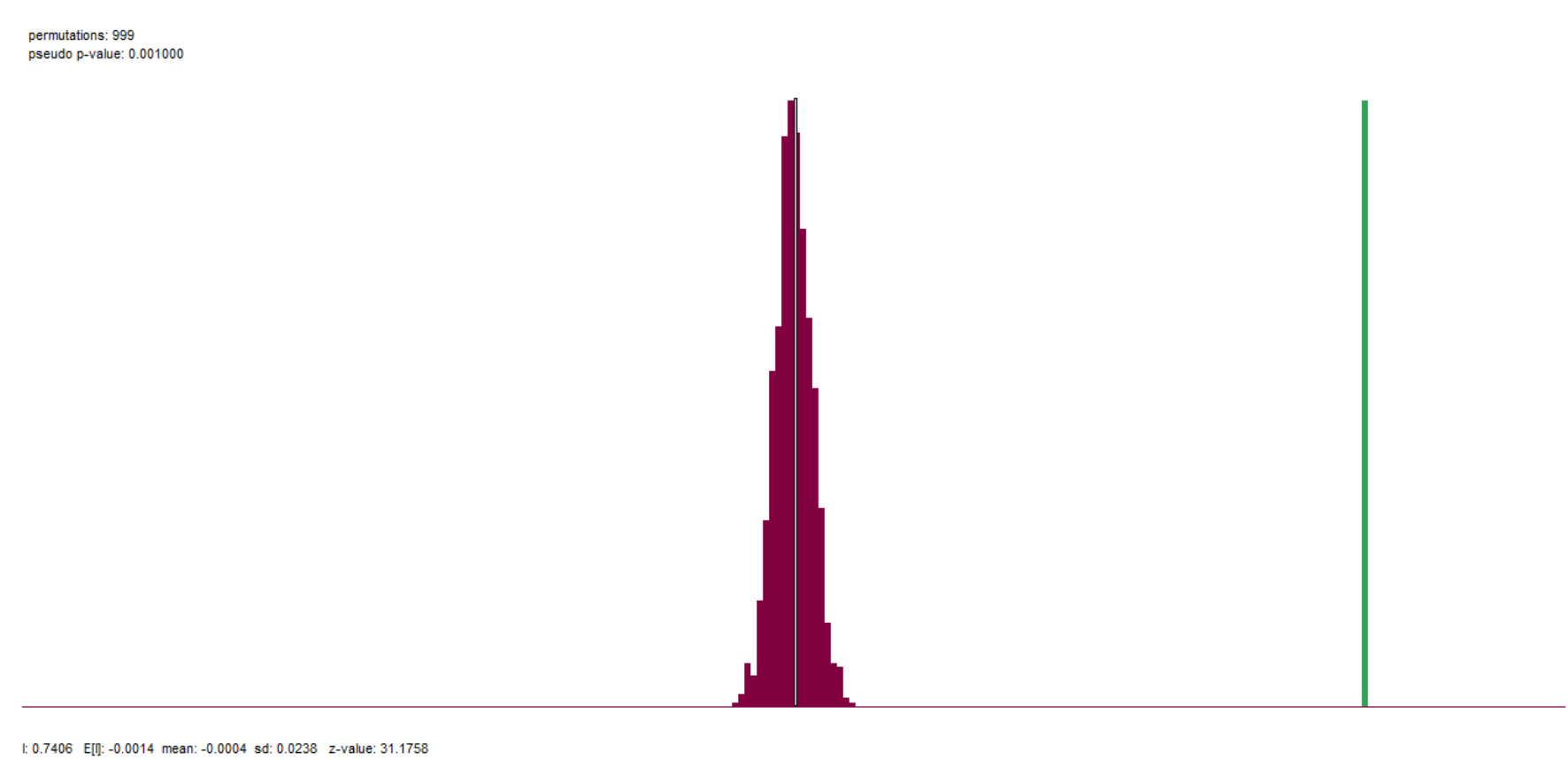

Supplement: Supplementary file 3 [file Datasheet1.zip › 6(B).png]

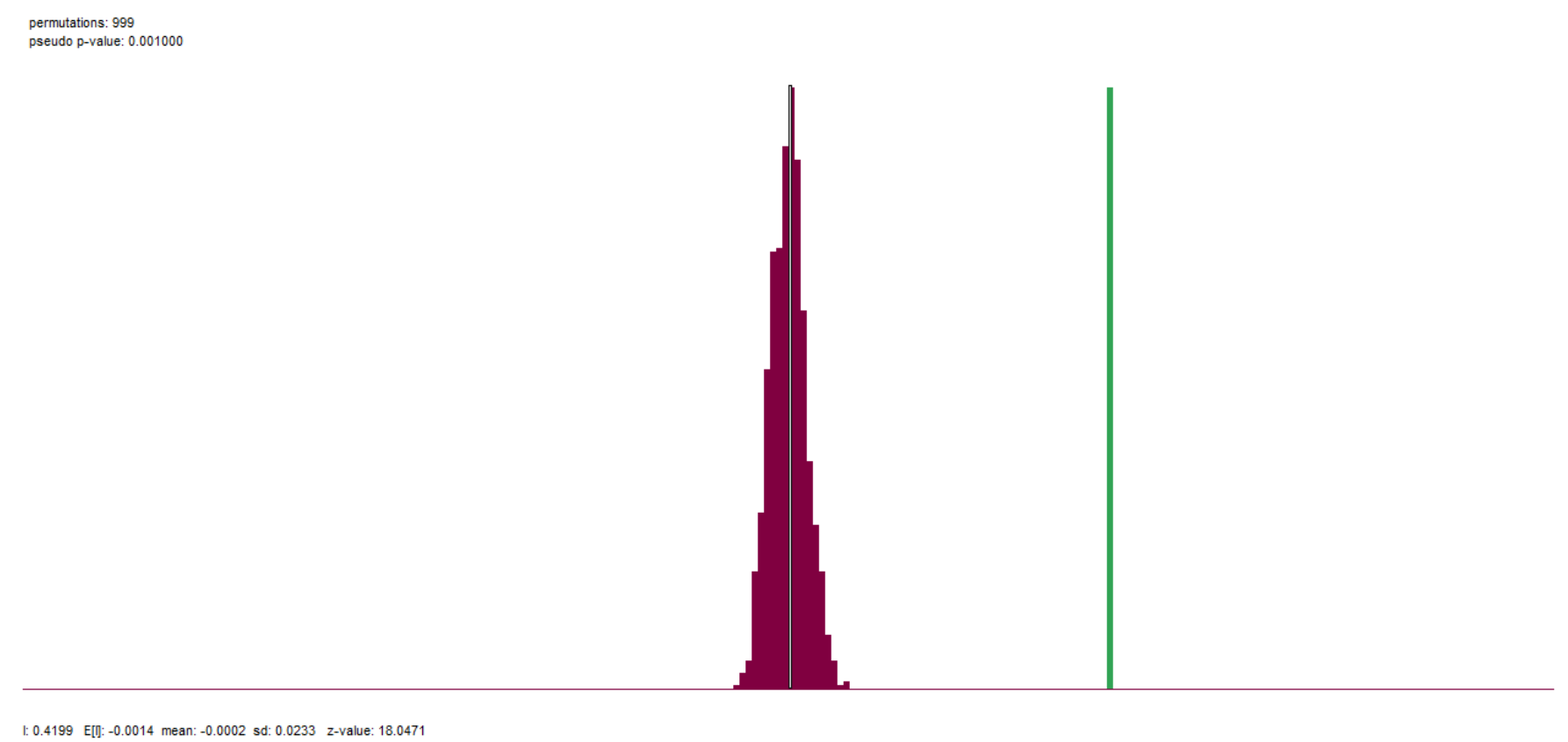

Supplement: Supplementary file 3 [file Datasheet1.zip › 6(C).png]

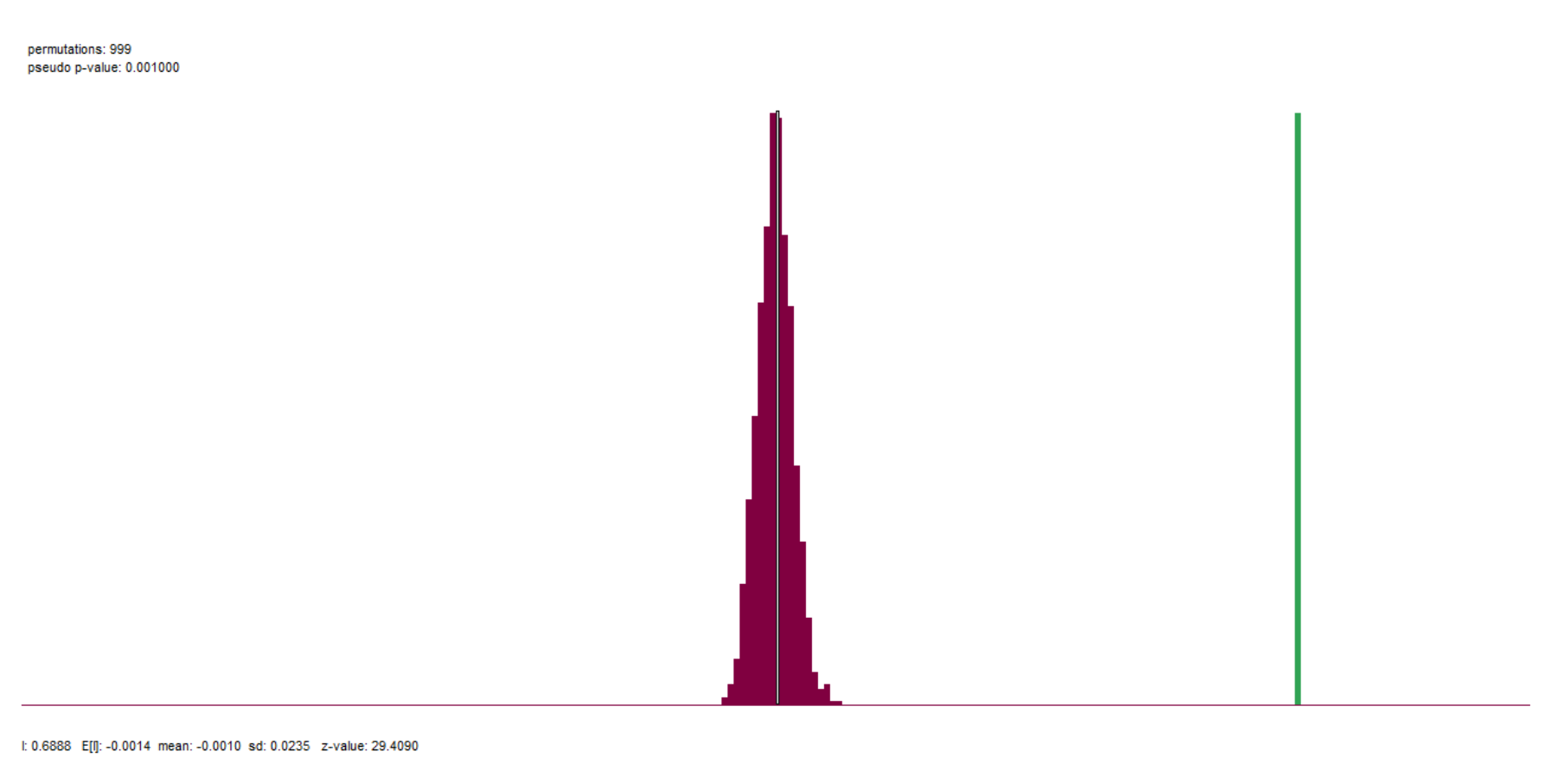

Supplement: Supplementary file 3 [file Datasheet1.zip › 6(D).png]
